# Influence of Fluorine Substitution on the Optical, Thermal, Electrochemical and Structural Properties of Carbazole-Benzothiadiazole Dicarboxylic Imide Alternate Copolymers

**DOI:** 10.3390/polym12122910

**Published:** 2020-12-04

**Authors:** Ary R. Murad, Ahmed Iraqi, Shujahadeen B. Aziz, Hunan Hi, Sozan N. Abdullah, M. A. Brza, Rebar T. Abdulwahid

**Affiliations:** 1 Department of Pharmaceutical Chemistry, College of Medical and Applied Sciences, Charmo University, Chamchamal, Sulaimani 46023, Iraq; ary.murad@charmouniversity.org; 2 Department of Chemistry, University of Sheffield, Sheffield S3 7HF, UK; a.iraqi@sheffield.ac.uk (A.I.); h.yi@sheffield.ac.uk (H.H.); 3 Hameed Majid Advanced Polymeric Materials Research Lab., Department of Physics, College of Science, University of Sulaimani, Qlyasan Street, Sulaimani 46001, Iraq; mohamad.brza@gmail.com (M.A.B.); rebar.abdulwahid@univsul.edu.iq (R.T.A.); 4 Department of Civil engineering, College of Engineering, Komar University of Science and Technology, Sulaimani 46001, Iraq; 5 Department of Chemistry, College of Science, University of Sulaimani, Qlyasan Street, Kurdistan Regional Government, Sulaimani 46001, Iraq; sozan.abdulla@univsul.edu.iq; 6 Department of Physics, College of Education, Old Campus, University of Sulaimani, Kurdistan Regional Government, Sulaimani 46001, Iraq

**Keywords:** conjugated copolymers, UV-vis study, Thermogravimetric analysis (TGA) analysis, X-ray diffraction (XRD) study, cyclic voltammetry (CV) analysis

## Abstract

In this work four novel donor-acceptor copolymers, PCDTBTDI-DMO, PCDTBTDI-8, P2F-CDTBTDI-DMO and P2F-CDTBTDI-8, were designed and synthesised via Suzuki polymerisation. The first two copolymers consist of 2,7-carbazole flanked by thienyl moieties as the electron donor unit and benzothiadiazole dicarboxylic imide (BTDI) as electron acceptor units. In the structures of P2F-CDTBTDI-DMO and P2F-CDTBTDI-8 copolymers, two fluorine atoms were incorporated at 3,6-positions of 2,7-carbazole to investigate the impact of fluorine upon the optoelectronic, structural and thermal properties of the resulting polymers. P2F-CDTBTDI-8 possesses the highest number average molecular weight (*M*_n_ = 24,200 g mol^−1^) among all the polymers synthesised. PCDTBTDI-DMO and PCDTBTDI-8 show identical optical band gaps of 1.76 eV. However, the optical band gaps of fluorinated copolymers are slightly higher than non-fluorinated counterparts. All polymers have deep-lying highest occupied molecular orbital (HOMO) levels. Changing the alkyl chain substituents on BTDI moieties from linear *n-*octyl to branched 3,7-dimethyloctyl groups as well as substituting the two hydrogen atoms at 3,6-positions of carbazole unit by fluorine atoms has negligible impact on the HOMO levels of the polymers. Similarly, the lowest unoccupied molecular orbital (LUMO) energy levels are almost comparable for all polymers. Thermogravimetric analysis (TGA) has shown that all polymers have good thermal stability and also confirmed that the fluorinated copolymers have higher thermal stability relative to those non-fluorinated analogues. Powder X-ray diffraction (XRD) studies proved that all polymers have an amorphous nature in the solid state.

## 1. Introduction

Due to a significant increase in energy demand, as well as the growing concern regarding the adverse impacts of using fossil energy resources, the necessity for a sustainable and renewable alternate source of energy has gained importance in recent years [1,2,3,4,5]. Photovoltaic technologies, which can directly harvest energy from sunlight, can be considered as a promising solution to address the growing global energy demand [6,7]. Among a wide range of technologies, photovoltaics (PVs) are one of the renewable resources that have potential qualities such as being clean, reliable and made from light material, having solution processability, low temperature manufacture and tunable electronic properties and being an affordable electricity system [8,9]. For the manufacturing of various PVs devices, different types of organic compounds such as conjugated polymers and polar polymers can be used. Conjugated polymers comprising alternating single and double bonds are one of the prominent materials that are used for optoelectronic applications [10]. The π-conjugated system gives rise to their semiconducting properties. Conducting polymers can be used in wide range of industrial applications including nonlinear optical devices, molecular electronics, organic solar cells (OSCs), organic field effect transistors (OFETs), organic light emitting diodes (OLEDs), memories and energy storage [11].

Carbazole is a fused-ring aromatic system, in which two benzene rings are fused in the center by a pyrrole ring. Carbazole is one of the most extensively utilized donor repeat units for optoelectronic applications, for example organic light emitting diodes (OLEDs) [12], organic field effect transistors (OFETs) [13,14] and solar cells [15]. It is structurally analogous to fluorene, however carbazole is a more electron rich unit due to its lone-pair of electrons on the nitrogen atom, which is involved in the aromatic system. In addition, carbazole derivatives have fully aromatic properties and they are able to form stable radical cations. They also have relatively high charge carrier mobilities and they show good photochemical and thermal stabilities [16].The nitrogen atom in the carbazole unit is easily functionalized with different alkyl chains for enhancing solubility of the resulting polymers [17,18].

Usually, carbazole moieties are linked through the 3,6-positions or 2,7-positions. Coupling of carbazole units through the 3,6-positions limits the π-conjugation of the resulting materials due to the large amount of twisting along the polymer backbone. However, connecting carbazole units via the 2,7-positions provides a greater degree of conjugation because the repeat units adopt a more planar structure [19,20]. However, cyclic voltammetry studies revealed that these types of polymers undergo irreversible oxidation and therefore they are less stable under electrolytic conditions. This is due to the nitrogen atom activating the 3,6-positions of carbazole, which results the formation of cross-linked polymers [21].

The stability of the polymer base of carbazole was studied by Iraqi and co-workers. It was found that by substituting hydrogen atoms with either methyl groups or fluorine atoms at the 3,6-positions of 2,7-linked carbazole, the resulting polymers show higher electrolytic stability relative to those non-substituted analogues [22,23].

Similar to fluorene, carbazole-based homopolymers usually have large band gaps, therefore these types of homopolymers are not suitable candidates for BHJ photovoltaic cells [15]. The band gap of polycarbazoles can be lowered by copolymerizing 2,7-carbazole moieties with a variety of acceptor units through alternating donor-acceptor (D-A) strategy. Based on this strategy, different types of high photovoltaic performance carbazole copolymers have been developed. The most successful D-A copolymer is poly[*N*-9′-heptadecanyl-2,7-carbazole-*alt*-5,5-(4′,7′-di-2-thienyl-2′,1′,3′-benzothiadiazole)] (PCDTBT), which was formed by copolymerizing 2,7-carbazole flanked by thienyl units as electron-rich moiety and benzothiadiazole (BT) as an acceptor unit. The photovoltaic devices based on PCDTBT:PC_61_BM showed a power-conversion efficiency (PCE) of 3.6% [24]. The performance of photovoltaic devices based on PCDTBT was further optimized up to 5.7% [25,26]. Later on, many research groups improved the efficiency of PCDTBT up to 6.79% by inserting different layers such as a titanium oxide (TiO_x_), conjugated polyelectrolyte, and alcohol/water soluble conjugated polymer between the active layer and top aluminum electrode [27,28,29]. A PCE of 7.5% was also reported for PCDTBT:PC_71_BM [30,31]. 

Zhang et al. reported on poly(2-(5-(5,6-bis(octyloxy)-4-(thiophen-2-yl)benzo[c][1,2,5]thiadiazol-7-yl)thiophen-2-yl)-9-octyl-9H-carbazole) (HXS-1) copolymer including 2,7-carbazole and 5,6-dialkoxy-2,1,3-benzothiadiazole [(OR)_2_BT] as an acceptor [32]. HXS-1 blended with PC_71_BM exhibited a PCE of 5.4%. Iraqi et al. designed and synthesised three copolymers, poly [9-(heptadecan-9-yl)-9*H*-carbazole-2,7-diyl-*alt*-(5,6-bis(octyloxy)-4,7-di(thiophen-2-yl)benzo[*c*][1,2,5]thiadiazole)-5,5-diyl] (PCDTBT-8), poly[9-(heptadecan-9-yl)-9*H*-carbazole-2,7-diyl-*alt*-(5,6-bis(octyloxy)-4,7-di(2,2′-bithiophen-5-yl)benzo[*c*][1,2,5]thiadiazole)-5,5-diyl] (PCDT2BT-8) and PCDTBT based on 2,7-carbazole flanked by thienyl or bithienyl units as donor units and BT or (OR)_2_BT as acceptor moieties. PCDTBT that blended with PC_71_BM delivered the highest PCE of 4.30% [33]. The same research group synthesised four new copolymers based on 2,7-carbazole flanked by thienyl or selenophenyl units as donor units and BT or (OR)_2_BT as acceptor moieties. PCDTBT fabricated with PC_71_BM in BHJ devices gave the highest PCE of 5.10% [34]. PCDT2BT-8 and Poly[9-(heptadecan-9-yl)-9H-carbazole-2,7-diyl-alt-(5,6-bis(octyloxy))-4,7-di(thieno[3,2-b]thiophen-2-yl)benzo[c][1,2,5]thiadiazole-5,5-diyl] (PCDTTBT-8) were also synthesised by Iraqi and co-workers using bithiophene and thieno[3,2-*b*]thiophene (TT) units as spacers between 2,7-carbazole and (OR)_2_BT. The BHJ solar cell based on PCDT2BT-8:PC_71_BM as active layer gave a PCE of 4.12%. However, PCDTTBT-8:PC_71_BM gave a higher PCE of 4.5% [35].

Although the physical and electronic properties of conjugated polymers are dominated by the selection of D and A units in the main chain of D-A copolymers, substituents such as fluorine atom can be used for fine tuning these properties. Incorporating fluorine atoms onto the polymer backbone could lower the HOMO energy level without introducing many steric effects [36]. The low-lying HOMO energy levels of fluorinated polymers can provide high open-circuit voltage (*V*_oc_) values in polymer solar cells (PSCs) [37]. You and co-workers have designed and synthesised a new acceptor unit, 4,7-di-2-thienyl-5,6-difluoro-2,1,3-benzothiadiazole (DTffBT). It was copolymerized with benzodithiophene (BDT) to build a PBDT-DTffBT copolymer. To study the influence of fluorine atoms on the efficiency of PSCs, they prepared PBDT-DTBT without fluorine atoms. PBDT-DTffBT has E_g_ of 1.7 eV, which is similar to that of PBDT-DTBT. However, the LUMO and HOMO levels of PBDT-DTffBT are lower relative to those of its non-fluorinated analogue. The PBDT-DTffBT fabricated into solar cell devices with PC_61_BM offered an impressive PCE of 7.2%. In contrast, the non-fluorinated counterpart only exhibited a PCE of 5.0% [38].

You et al. further developed another new acceptor unit based on 2-alkyl-2′,1′,3′-benzotriazole (TAZ) by replacing two hydrogen atoms at 5,6-positions of TAZ with two fluorine atoms to build a FTAZ. It was copolymerized with a BDT to generate a novel PBDT-FTAZ copolymer. Moreover, they prepared PBDT-HTAZ copolymer without fluorine atoms to investigate the impact of fluorine atoms on the performance of the devices. BHJ photovoltaic devices composed of PBDT-FTAZ and PC_61_BMas active layer delivered a remarkable PCE of 7.1%. On the other hand, PBDT-HTAZ showed only a PCE of 4.36% [36].

In 2013, Wang et al. reported three copolymers based on BDT and DTffBT with different solubilizing chains, PBDT_TEH_-DT_H_ffBT, PBDT_TEH_-DT_EH_ffBT and PBDT_HDO_-DT_H_ffBT. PBDT_TEH_-DT_H_ffBT and PBDT_TEH_-DT_EH_ffBT blended with PC_71_BM delivered the PCE of 4.46 and 6.20%, respectively. On the other hand, PBDT_HDO_-DT_H_ffBT offered an unprecedentedly high PCE of 8.30% [39]. Peng et al. developed a new acceptor moiety based on DTBT by substituting one hydrogen atom at 5-position of BT with fluorine atom to construct DTfBT. It was copolymerized with alkylthienyl substituted BDT to form PBDT-DTfBT. The BHJ solar cell based on PBDT-DTfBT:PC_71_BM showed a high PCE of 6.21% [40].

Jen and co-workers reported a PCPDTFBT copolymer including cyclopentadithiophene (CPDT) and partially fluorinated BT (FBT). In parallel, they synthesised the non-fluorinated PCPDTBT counterpart to examine the effect of fluorine on the photovoltaic efficiency of the device. The PCPDTFBT:PC_71_BM delivered much higher PCE than PCPDTBT (5.51 vs. 2.75%) [41]. Nehr et al. independently reported the same copolymers PCPDTFBT and PCPDTBT. PCPDTFBT and PCPDTBT fabricated with PC_71_BM delivered a PCE of 6.16% and 3.59% respectively [42].

For all of the highly efficient copolymers mentioned above, fluorine was incorporated on the acceptor units. Bo and co-workers inserted fluorine atoms on the donor unit. They reported a novel copolymer poly[3,6-difluoro-9-octyl-9H-2,7-carbazole-alt-5,5-(4′,7′-di-2-thienyl-5′,6′-bis(octyloxy)-2′,1′,3′-benzothiadiazole)] (O-PDFCDTBT) based on 3,6-difluoro-2,7-carbazole flanked by thienyl units and (OR)_2_BT. The copolymer had a low molecular weight (*M*_n_ = 9100 g mol^−1^) due to its limited solubility. The BHJ solar cell based on O-PDFCDTBT:PC_71_BM as the active layer showed a PCE of 4.8% [43]. They replaced the *n*-octyl side chains on carbazole units by 2-hexyldecyl side chains (HD) so as to increase the solubility and molecular weight of the resulting polymer. The new copolymer, HD-PDFCDTBT was synthesised with a *M*_n_ as high as 38,000 g mol^−1^. The HD-PDFCDTBT showed much higher hole mobility than O-PDFCDTBT, probably due to the higher molecular weight of the former polymer. HD-PDFCDTBT blended with PC_71_BM delivered a high PCE of 7.39% [44].

The extensive use of electronic devices with increased energy demand has a negative impact on the environment. The electronic wastes and greenhouse gases from energy production sources are causing serious global environmental issues and climate change. Through governing several properties of biodegradable polymer, a desired environmentally friendly material for energy devices can be viable. Conductive or alternating polymers with small band gaps are crucial for optoelectronic device applications including organic solar cells. This research will emphasis different properties of some modified copolymers with reduced band gap and highlights the possibility of using those copolymers in energy devices. In this work four novel alternating copolymers, PCDTBTDI-DMO, PCDTBTDI-8, P2F-CDTBTDI-DMO and P2F-CDTBTDI-8 were designed and synthesised via Suzuki polymerisation. The structural, thermal, electrochemical and optical properties were studied. The result of the present work indicates that copolymerization is a novel approach to synthesizing conductive or alternating polymers with low band gaps and amorphous phases.

## 2. Experimental Section

### 2.1. Materials

All of the starting materials and reagents were obtained from Sigma-Aldrich (Gillingham, UK) and Alfa Aesar (Heysham, UK) and utilized without further purification. The majority of the reactions were carried out under argon atmosphere. Anhydrous solvents used for the reactions were obtained from Grubbs solvent purification system within the Sheffield University/Chemistry Department. All the monomers used for preparing the polymers in this article were synthesised according to the procedure part.

### 2.2. Measurements

All ^1^H NMR and ^13^C NMR nuclear magnetic resonance (NMR) spectra for the monomers were measured either with a Bruker Avance AV 3HD 400 (400 MHz) spectrometer (Bruker, Berlin, Germany) in deuterated chloroform (CDCl_3_), deuterated acetone (CD_3_COCD_3_) or deuterated dimethyl sulfoxide (CD_3_SOCD_3_) as the solvents at room temperature. The ^1^H NMR spectra for the polymers were measured with a Bruker AV 3HD 500 (500 MHz) (Bruker, Berlin, Germany) in deuterated 1,1,2,2-tetrachloroethane(C_2_D_2_Cl_4_) as the solvent at 100 °C. The chemical shifts were measured in parts per million (ppm). The coupling constants (*J*) are calculated in Hertz (Hz). The ^1^H NMR and ^13^C NMR spectra were analysed using Bruker TopSpin 3.2 software. Elemental analysis (CHN) was performed by either the Perkin Elmer 2400 CHNS/O Series II Elemental Analyzer (Horiba, Northampton, UK) or Vario MICRO Cube CHN/S Elemental Analyzer (Eltra, Chester, UK) for CHN analysis. Anion analysis (Br, I and S) was performed by the Schöniger oxygen flask combustion method. Mass spectra for the monomers were recorded on Agilent 7200 accurate mass Q-TOF GC-MS spectrometer (Agilent, Santa Clara, CA, United State). Helium was used as a carrier gas at a rate of (1.2 mL min^−1^), the injection volume was (1.0 μL) and the concentration of measured sample was (5 mg mL^−1^) in CHCl_3_ solvent. The temperature program was between 60 to 320 °C at 10 °C min^−1^. Mass spectra for the monomers were obtained by the electron ionization method (EI). Gel permeation chromatography (GPC) measurements accomplished by Viscotek GPC Max (Malvern Panalytical, Malvern, UK), a waters 410 instrument with a differential refractive index detector, two Polymer Labs PLgel 5μ Mixed C (7.5 × 300 mm) columns and a guard (7.5 × 50 mm). Molecular weights for the polymers were determined by preparing polymer solutions (2.5 mg mL^−1^) using HPLC grade CHCl_3_. The columns were thermostated at 40 °C using CHCl_3_. UV-vis absorption spectra were measured by SPECORD S600 UV/visible Spectrophotometer (Hach, Dusseldorf, Germany) at room temperature. The absorbance of the polymers was measured in CHCl_3_ solution using quartz cuvettes (light path length = 10 mm) and blank quartz cuvettes including CHCl_3_ was used as a reference. The polymers were coated on quartz substrates from CHCl_3_ solutions (1 mg mL^−1^) and blank quartz substrate was used as a reference. Thermogravimetric analysis (TGA) measurements were recorded by Perkin Elmer (Pyris 1) thermogravimetric Analyser. Platinum pans were used as sample holders and the weight of the measured samples was about (3 mg). Cyclic voltammograms were measured using a Model 263A Potentiostat/Galvanostat-Princeton Applied Research (Champaign, IL, USA). A standard three electrode system was used based on a Pt disk working electrode, a silver wire reference electrode (Ag/Ag^+^) inserted in (0.01 M) AgNO_3_ solution in acetonitrile and put it in the electrolyte solution and a Pt wire counter electrode was purged with argon atmosphere during all measurements at room temperature. Tetrabutylammonium perchlorate in acetonitrile (Bu_4_NClO_4_/CH_3_CN) (0.1 M) was used as the electrolyte. Polymer thin films were drop cast onto the Pt disk from polymer solutions in CH_3_Cl (1 mg mL^−1^) and dried under nitrogen prior to measurement. Ferrocene (Fc/Fc^+^) was used as a reference redox system. Powder X-ray diffraction (XRD) for the polymers was measured by Bruker D8 ADVANCE X-ray powder diffractometer (Bruker, Berlin, Germany). Infrared absorption spectra were recorded on ATR Perkin Elmer Rx/FT-IR system (PerkinElmer, Bucks, UK) and Nicolet Model 205 FT-IR spectrometer (Nicolet instrument, Sainte-Julie, QC, Canada).

### 2.3. Monomers and Polymers Synthesis

**Synthesis of 2,5-dibromothiophene (1):** Thiophene (25.00 g, 297.12 mmol) in DMF (250 mL) was added to a flask and cooled to −15 °C. To this solution, NBS (110.00 g, 618.04 mmol) in DMF (300 mL) was added dropwise in the dark and the reaction stirred overnight at RT. The reaction contents was put into ice and DCM and subsequently extracted with DCM and the organic phase washed with deionized H_2_O to a neutral pH. The organic layer collected and dried over MgSO_4_ and the solvent concentrated to afford the product which was purified by vacuum distillation and gave 1 as a yellow oil (59.30 g, 245 mmol, 82% yield) [45]. ^1^H NMR (CDCl_3_, δ): 6.87 (s, 2H). ^13^C NMR (CDCl_3_, δ): 130.4, 111.6. FT-IR (cm^−1^): 3096, 1726, 1516, 1410, 1200. EI-MS (*m*/*z*): 242 [M]^+^. EA (%) calculated for C_4_H_2_Br_2_S: C, 19.86; H, 0.83; Br, 66.06, S, 13.25. Found: C, 20.01; H, 0.85; Br, 65.02, S, 11.96.

**Synthesis of 2,5-dibromo-3,4-dinitrothiophene (2):** Concentrated H_2_SO_4_ (150 mL) and fuming H_2_SO_4_ (150 mL, 20% free SO_3_) were combined in a flask. This flask was cooled to 0 °C and 1 (26.00 g, 107.46 mmol) was added dropwise. Concentrated nitric acid (125 mL) was added dropwise and the reaction contents kept under 20 °C. During the addition of nitric acid yellow precipitate formed quickly. The mixture was stirred for 3h at 20–30 °C. Then, the mixture was poured into ice and upon melting of the ice a yellow precipitate filtrated and was washed thoroughly with deionized H_2_O. The product recrystallized from methanol to afford 2 as yellow crystals (32.50 g, 98 mmol, 91% yield) [46]. ^13^C NMR (CDCl_3_, δ): 140.7, 113.4. FT-IR (cm^−1^): 2886, 2851, 2813, 1535, 1497, 1345, 1081. EI-MS (*m*/*z*): 332 [M]^+^. EA (%) calculated for C_4_Br_2_N_2_O_4_S: C, 14.47; N, 8.44; S, 9.66; Br, 48.15. Found: C, 14.51; N, 7.91; S, 9.19; Br, 46.57.

**Synthesis of 3′,4′-dinitro-2,2′:5′,2″-terthiophene (3):** In a flask, 2 (9.90 g, 29.82 mmol), 2-(tributylstannyl)thiophene (27.82 g, 74.54 mmol) and PdCl_2_(PPh_3_)_2_ (0.45 g, 0.64 mmol) were added. The system degassed under argon, anhydrous toluene (100 mL) was added and heated at 115 °C for 24 h. The flask cooled to RT and the volatiles removed to obtain the product which purified by column chromatography with gradient (petroleum ether, 0%–50% DCM) to obtain an orange solid and the product was further purified by recrystallization from methanol to afford 3 as orange crystals (9.10 g, 27 mmol, 90% yield) [47]. ^1^H NMR (CDCl_3_, δ): 7.62 (dd, 2H, *J* = 1.0 Hz, 5.0 Hz), 7.56 (dd, 2H, *J* = 1.0 Hz, 4.0 Hz), 7.19 (dd, 2H, *J* = 4.0 Hz, 5.0 Hz). ^13^C NMR (CDCl_3_, δ): 135.9, 133.9, 131.3, 131.2, 128.4, 128.0. FT-IR (cm^−1^): 3076, 1821, 1528, 1379, 1348, 1299, 1223, 1066. EI-MS (*m*/*z*): 338 [M]^+^. EA (%) calculated for C_12_H_6_N_2_O_4_S_3_: C, 42.60; H, 1.79; N, 8.28; S, 28.42. Found: C, 42.49; H, 1.66; N, 8.13; S, 28.16.

**Synthesis of 3′,4′-diamino-2,2′:5,2″-terthiophene (4):** EtOH (31 mL) and HCl (62 mL, 35%) were added to 3 (3.00 g, 8.86 mmol) in a flask. To this mixture, anhydrous tin(II) chloride (31.00 g, 163.50 mmol) in ethanol (62 mL) was added and stirred at 30 °C for 24h. The mixture cooled to RT and was put into cold NaOH. To this mixture, toluene was added and then stirred vigorously and filtered through celite. The product was extracted with toluene and the organic phases washed with NaCl and subsequently dried over anhydrous MgSO_4_. The solvent concentrated to obtain 4 as a brown solid (2.40 g, 9 mmol, 97% yield) [48]. ^1^H NMR (CDCl_3_, δ): 7.30 (d, 2H, *J* = 2.0 Hz), 7.27 (s, 2H), 7.09–7.14 (m, 2H), 3.76 (bs, 4H). ^13^C NMR (CDCl_3_, δ): 136.0, 133.6, 127.8, 124.0, 124.0, 110.1. FT-IR (cm^−1^): 3371, 3298, 3224, 3182, 3096, 1631, 1615, 1573, 1528, 1509, 1441, 1336, 1294, 1070. EI-MS (*m*/*z*): 278 [M]^+^. EA (%) calculated for C_12_H_10_N_2_S_3_: C, 51.77; H, 3.62; N, 10.06; S, 34.55. Found: C, 51.69; H, 3.54; N, 9.97; S, 34.78.

**Synthesis of 4,6-bis(2-thienyl)-thieno[3,4-*c*][1,2,5]-thiadiazole (5):** 4 (1.67 g, 5.99 mmol) was dissolved in dry pyridine (30 mL) in a flask and degassed under argon. To this mixture, *N*-thionylaniline (1.60 g, 11.49 mmol) was added dropwise and chlorotrimethylsilane (4.50 g, 41.42 mmol) then added dropwise, resulting in a dark blue colour. The reaction contents were stirred for 3 h at RT and then put into DCM. The solution was washed with HCl and with deionized water and extracted with DCM. The organic phase dried over anhydrous MgSO_4_ and was subsequently filtered. The solvent evaporated to afford the product which purified via chromatography with DCM to afford 5 as blue crystals (1.72 g, 6 mmol, 93% yield) [49]. ^1^H NMR (CDCl_3_, δ): 7.59 (dd, 2H, *J* = 1.0 Hz, 3.5 Hz), 7.34 (dd, 2H, *J* = 1.0 Hz, 5.0 Hz), 7.12 (dd, 2H, *J* = 3.5 Hz, 5.0 Hz). ^13^C NMR (CDCl_3_, δ): 156.3, 135.0, 128.2, 125.4, 124.3, 112.4. FT-IR (cm^−1^): 3102, 3073, 1797, 1525, 1483, 1365, 1223, 1137, 1047. EI-MS (*m*/*z*): 306 [M]^+^. EA (%) calculated for C_12_H_6_N_2_S_4_: C, 47.04; H, 1.97; N, 9.14; S, 41.85. Found: C, 47.25; H, 2.18; N, 8.83; S, 39.16.

**Synthesis of 4,7-di(thien-2-yl)-2,1,3-benzothiadiazole-5,6-dimethyl ester (6):** 5 (1.86 g, 6.06 mmol) and dimethyl acetylenedicarboxylate (1.73 g, 12.17 mmol) were combined in a flask. The system was evacuated and refilled with argon for three cycles before anhydrous xylene (40 mL) was added. The reaction contents refluxed for 24 h. The flask cooled to RT and the solvent was removed to afford the product which was purified by column chromatography with gradient (petroleum ether, 0–50% DCM) to afford 6 as yellow crystals (2.37 g, 6 mmol, 94% yield) [50]. ^1^H NMR (CDCl_3_, δ): 7.62 (dd, 2H, *J* = 1.0 Hz, 5.0 Hz), 7.44 (dd, 2H, *J* = 1.0 Hz, 3.5 Hz), 7.22 (dd, 2H, *J* = 3.5 Hz, 5.0 Hz), 3.78 (s, 6H). ^13^C NMR (CDCl_3_, δ): 168.1, 153.6, 135.1, 132.0, 129.7, 129.0, 127.3, 126.2, 53.1. FT-IR (cm^−1^): 3109, 2975, 2932, 2900, 2865, 2159, 2031, 1971, 1730, 1513, 1460, 1318, 1283, 1198. EI-MS (*m*/*z*): 416 [M]^+^. EA (%) calculated for C_18_H_12_N_2_O_4_S_3_: C, 51.91; H, 2.90; N, 6.73; S, 23.09. Found: C, 51.86; H, 2.94; N, 6.61; S, 22.97.

**Synthesis of 4,7-di(thien-2-yl)-2,1,3-benzothiadiazole-5,6-dicarboxylic acid (7):** Sodium hydroxide (4.00 g, 100.00 mmol) was dissolved in deionized water (30 mL) and added to a flask. To this solution, ethanol (200 mL) and 6 (2.27 g, 5.45 mmol) were added and the reaction contents refluxed for 24 h. The flask cooled to RT and deionized H_2_O was added. This mixture cooled to 0 °C and was neutralised by HCl to precipitate the product. The precipitate was filtered and subsequently washed with deionized H_2_O. The precipitate was dried under high vacuum to afford 7 as yellow solid (1.80 g, 5 mmol, 85% yield) [51]. ^1^H NMR (CD_3_SOCD_3_, δ): 7.86 (dd, 2H, *J* = 1.0 Hz, 5.0 Hz), 7.47 (dd, 2H, *J* = 1.0 Hz, 3.5 Hz), 7.25 (dd, 2H, *J* = 3.5 Hz, 5.0 Hz). ^13^C NMR (CD_3_SOCD_3_, δ): 168.4, 152.5, 134.8, 133.0, 129.7, 129.3, 127.2, 123.8. FT-IR (cm^−1^): 3106, broad (3300-2600), 2162, 2024, 1971, 1815, 1765, 1705, 1552, 1453, 1386, 1261, 1152, 1020. EI-MS (*m*/*z*): 387 [M-H]^+^. EA (%) calculated for C_16_H_8_N_2_O_4_S_3_: C, 49.48; H, 2.08; N, 7.21; S, 24.76. Found: C, 45.33; H, 2.70; N, 6.47; S, 21.35.

**Synthesis of 4,7-di(thien-2-yl)-2,1,3-benzothiadiazole-5,6-dicarboxylic anhydride (8):** 7 (1.15 g, 2.96 mmol) and anhydrous acetic anhydride (10.00 g, 97.95 mmol) were combined in a flask. The system was evacuated and refilled with argon for three cycles before anhydrous xylene (30 mL) was added. The mixture was heated at 130 °C for 6 h. The mixture cooled to RT and the solvent evaporated to obtain 8 as red solid (1.06 g, 3 mmol, 97% yield) [52]. ^1^H NMR (CDCl_3_, δ): 8.11 (dd, 2H, *J* = 1.0 Hz, 4.0 Hz), 7.82 (dd, 2H, *J* = 1.0 Hz, 5.0 Hz), 7.33 (dd, 2H, *J* = 4.0 Hz, 5.0 Hz). ^13^C NMR (CD_3_SOCD_3_, δ): 162.0, 156.0, 134.3, 132.6, 131.4, 127.8, 127.6, 125.5. FT-IR (cm^−1^): 3131, 3109, 3081, 1808, 1765, 1552, 1453, 1393, 1247, 1152, 1088. EI-MS (*m*/*z*): 370 [M]^+^. EA (%) calculated for C_16_H_6_N_2_O_3_S_3_: C, 51.88; H, 1.63; N, 7.56; S, 25.97. Found: C, 52.11; H, 2.00; N, 7.20; S, 24.55.

**Synthesis of 3,7-dimethyloctyl bromide (9)**: Triphenylphosphine (21.10 g, 80.44 mmol) was added to a mixture of 3,7-dimethyloctyl alcohol (12.61 g, 79.69 mmol) and dichloromethane (250 mL) and stirred in a flask. To this mixture, NBS (14.26 g, 80.14 mmol) was added portionwise and stirred at RT for 90 min. The mixture was washed with NaHCO_3_ solution, dried over anhydrous MgSO_4_, filtered and the solvent evaporated. The substance was stirred in petroleum ether for 1h at RT, filtered and the filtrate evaporated. The product was purified by chromatography with petroleum ether to yield 9 as colourless oil (23.00 g, 59 mmol, 73% yield) [53]. ^1^H NMR (CDCl_3_, δ): 3.55–3.37 (m, 2H), 1.96–1.83 (m, 1H), 1.77–1.61 (m, 2H), 1.60–1.49 (m, 1H), 1.41–1.24 (m, 3H), 1.22–1.11 (m, 3H), 0.82–0.94 (m, 9H). ^13^C NMR (CDCl_3_, δ): 40.1, 39.2, 36.7, 32.3, 31.7, 28.0, 24.6, 22.7, 22.6, 19.0. FT-IR (cm^−1^): 2953, 2925, 2868, 1464, 1382, 1261, 1173. EI-MS (*m*/*z*): 222.1 [M]^+^. EA (%) calculated for C_10_H_21_Br: C, 54.30; H, 9.57; Br, 36.13. Found: C, 55.04; H, 9.53; Br, 34.23.

**Synthesis of *N*-(3,7-dimethyloctyl)phthalimide (10):** 9 (4.07 g, 18.40 mmol) and anhydrous DMF (20 mL) were added into a flask. To this mixture, potassium phthalimide (3.75 g, 20.27 mmol) was added and the reaction contents heated to 90 °C for 17 h. The mixture cooled to RT and was put in deionized H_2_O and the product subsequently extracted with DCM. The organic extracts were combined and washed with KOH and deionised water. The organic phase dried over anhydrous MgSO_4_ and the solvent evaporated to obtain the product which purified via chromatography with dichloromethane to yield 10 as colourless oil (5.29 g, 18 mmol, 91% yield) [54]. ^1^H NMR (CDCl_3_, δ): 7.85 (dd, 2H, *J* = 3.0 Hz, 5.5 Hz), 7.72 (dd, 2H, *J* = 3.0 Hz, 5.5 Hz), 3.80–3.66 (m, 2H), 1.77–1.66 (m, 1H), 1.53–1.43 (m, 3H), 1.41–1.25 (m, 3H), 1.20–1.11 (m, 3H), 0.98 (d, 3H, *J* = 6.5 Hz), 0.87 (d, 6H, *J* = 7.0 Hz). ^13^C NMR (CDCl_3_, δ): 168.4, 133.8, 132.2, 123.1, 39.2, 37.0, 36.3, 35.5, 30.7, 27.9, 24.5, 22.7, 22.6, 19.4. FT-IR (cm^−1^): 2953, 2925, 2868, 1772, 1706, 1616, 1469, 1398, 1267, 1189, 1055. EI-MS (*m*/*z*): 288.2 [MH]^+^. EA (%) calculated for C_18_H_25_NO_2_: C, 75.22; H, 8.77; N, 4.87. Found: C, 72.17; H, 8.62; N, 4.43.

**Synthesis of 3,7-dimethyl-1-octanamine (11):** 10 (6.03 g, 20.98 mmol), hydrazine hydrate (4.0 mL, 65.0 mmol, 51%) and methanol (100 mL) were combined in a flask. The reaction contents refluxed until the starting material disappeared. Upon completion, excess HCl was added and the mixture refluxed for 1h and then cooled to RT. The precipitate was filtered and washed with water. The methanol concentrated and the residue was diluted with dichloromethane. The organic layer was washed with KOH and the product was extracted with dichloromethane. The organic phase was washed with NaCl, dried over anhydrous MgSO_4_ and the solvent concentrated to yield 11 as a brown oil (2.85 g, 18 mmol, 86% yield) [55]. ^1^H NMR (CDCl_3_, δ): 2.82–2.62 (m, 2H), 1.60–1.43 (m, 3H), 1.35–1.22 (m, 4H), 1.20–1.06 (m, 3H), 0.88 (dd, 9H, *J* = 2.0 Hz, 6.5 Hz). ^13^C NMR (CDCl_3_, δ): 41.1, 40.1, 39.3, 37.3, 30.5, 28.0, 24.7, 22.7, 22.6, 19.6. FT-IR (cm^−1^): 3521, 3375, 3219, 3021, 2953, 2925, 2868, 2155, 2028, 1978, 1598, 1464, 1382, 1166, 1063. EI-MS (*m*/*z*): 157.2 [M]^+^. EA (%) calculated for C_10_H_23_N: C, 76.36; H, 14.74; N, 8.90. Found: C, 71.74; H, 13.51; N, 7.71.

**Synthesis of 4,7-di(thien-2-yl)-2,1,3-benzothiadiazole-5,6-*N*-(3,7-dimethyloctyl)dicarboxylic imide (12):** 8 (1.00 g, 2.69 mmol), acetic acid (50 mL, 100%) and 11 (0.88 g, 5.59 mmol) were combined in a flask. The system was evacuated and refilled with argon for three cycles and heated at 110 °C overnight. The mixture cooled to RT, acetic anhydride (20 mL) was added and heated at 110 °C for 6 h. The mixture cooled to RT and the solvent concentrated to yield the product which was purified by chromatography with (60:10, petroleum ether: ethyl acetate) to afford 12 as an orange solid (1.15 g, 2.3 mmol, 84% yield) [52]. ^1^H NMR (CDCl_3_, δ): 7.91 (dd, 2H, *J* = 1.0 Hz, 3.5 Hz), 7.73 (dd, 2H, *J* = 1.0 Hz, 5.0 Hz), 7.30 (dd, 2H, *J* = 3.5 Hz, 5.0 Hz), 3.84–3.70 (m, 2H), 1.78–1.65 (m, 1H), 1.55–1.43 (m, 3H), 1.39–1.22 (m, 3H), 1.20–1.08 (m, 3H), 0.97 (d, 3H, *J* = 6.0 Hz), 0.86 (d, 6H, *J* = 6.0 Hz). ^13^C NMR (CDCl_3_, δ): 165.7, 156.5, 133.1, 131.5, 130.2, 127.0, 126.9, 126.7, 39.2, 37.2, 37.0, 35.2, 31.0, 27.9, 24.6, 22.7, 22.6, 19.4. FT-IR (cm^−1^): 3439, 3102, 3074, 2953, 2925, 2865, 1804, 1751, 1694, 1549, 1453, 1364, 1226, 1162, 1056. EI-MS (*m*/*z*): 510.1 [MH]^+^. EA (%) calculated for C_26_H_27_N_3_O_2_S_3_: C, 61.27; H, 5.34; N, 8.24; S, 18.87. Found: C, 61.59; H, 5.56; N, 7.94; S, 16.79.

**Synthesis of 4,7-di(thien-2-yl)-2,1,3-benzothiadiazole-5,6-*N*-octyl-dicarboxylic imide (13):** 13 was prepared following the same procedure for synthesis of 12 except *N*-octylamine (1.20 g, 9.28 mmol) was used. 13 was obtained as an orange solid (1.20 g, 2.5 mmol, 93% yield) [52]. ^1^H NMR (CDCl_3_, δ): 7.91 (dd, 2H, *J* = 1.0 Hz, 3.5 Hz), 7.73 (dd, 2H, *J* = 1.0 Hz, 5.0 Hz), 7.30 (dd, 2H, *J* = 3.5 Hz, 5.0 Hz), 3.74 (t, 2H, *J* = 7.5 Hz), 1.65-1.76 (m, 2H), 1.23–1.41 (m, 10H), 0.88 (t, 3H, *J* = 7.0 Hz). ^13^C NMR (CDCl_3_, δ): 165.8, 156.5, 133.1, 131.5, 130.2, 127.1, 126.9, 126.7, 39.0, 31.8, 29.1, 28.2, 27.0, 22.7, 14.0. FT-IR (cm^−1^): 3443, 3102, 3070, 2918, 2854, 1808, 1754, 1694, 1556, 1457, 1364, 1226, 1169, 1098. EI-MS (*m*/*z*): 481.1 [M]^+^. EA (%) calculated for C_24_H_23_N_3_O_2_S_3_: C, 59.85; H, 4.81; N, 8.72; S, 19.97. Found: C, 59.91; H, 4.93; N, 8.70; S, 20.72.

**Synthesis of 4,7-di(5-bromo-thien-2-yl)-2,1,3-benzothiadiazole-5,6-*N*-(3,7-dimethyloctyl)dicarboxylic imide (M1):** 12 (1.00 g, 1.96 mmol) and THF (100 mL) were combined in a flask. To this mixture, NBS (1.74 g, 9.77 mmol) was added and stirred at RT overnight in the dark. The solvent evaporated to obtain the product as a red solid, subsequently washed with cold CH_3_OH, filtered and dried. The product was purified via chromatography with DCM to yield M1 as a red solid (1.28 g, 2 mmol, 98% yield) [51]. ^1^H NMR (CDCl_3_, δ): 7.80 (d, 2H, *J* = 4.0 Hz), 7.24 (d, 2H, *J* = 4.0 Hz), 3.70–3.84 (m, 2H), 1.78–1.66 (m, 1H), 1.54–1.44 (m, 3H), 1.41–1.22 (m, 3H), 1.20–1.11 (m, 3H), 0.98 (d, 3H, *J* = 6.0 Hz), 0.87 (d, 6H, *J* = 6.5 Hz). ^13^C NMR (CDCl_3_, δ): 165.6, 155.9, 134.1, 133.0, 129.8, 126.4, 125.8, 118.7, 39.2, 37.3, 37.0, 35.2, 31.0, 27.9, 24.6, 22.7, 22.6, 19.4. FT-IR (cm^−1^): 3429, 3120, 2957, 2918, 2865, 1747, 1691, 1563, 1460, 1364, 1283, 1073. EI-MS (*m*/*z*): 666.9 [M]^+^. EA (%) calculated for C_26_H_25_Br_2_N_3_O_2_S_3_: C, 46.78; H, 3.78; Br, 23.94; N, 6.30; S, 14.41. Found: C, 46.61; H, 3.61; Br, 23.95; N, 6.29; S, 14.64.

**Synthesis of 4,7-di(5-bromo-thien-2-yl)-2,1,3-benzothiadiazole-5,6-*N*-octyl-dicarboxylic imide (M2):** M2 was prepared following the same procedure for synthesis of M1, where compound 13 (1.00 g, 2.07 mmol) was used with THF (100 mL) and NBS (1.84 g, 10.33 mmol). M2 was obtained as red solid (1.27 g, 2 mmol, 96% yield) [51]. ^1^H NMR (CDCl_3_, δ): 7.80 (d, 2H, *J* = 4.0 Hz), 7.24 (d, 2H, *J* = 4.0 Hz), 3.75 (t, 2H, *J* = 7.0 Hz,), 1.66–1.75 (m, 2H), 1.23–1.40 (m, 10H), 0.88 (t, 3H, *J* = 6.5 Hz,). ^13^C NMR (CDCl_3_, δ): 165.7, 156.0, 134.1, 133.0, 129.8, 126.4, 125.9, 118.7, 39.0, 31.8, 29.1, 28.3, 27.0, 22.6, 14.1. FT-IR (cm^−1^): 3421, 3120, 2953, 2911, 2850, 1744, 1687, 1556, 1446, 1375, 1244, 1176. EI-MS (*m*/*z*): 638.9 [M]^+^. EA (%) calculated for C_24_H_21_Br_2_N_3_O_2_S_3_: C, 45.08; H, 3.31; N, 6.57; S, 15.04; Br, 24.99. Found: C, 44.79; H, 3.41; N, 6.47; S, 15.74; Br, 28.80.

**Synthesis of 1,4-dibromo-2-fluoro-5-nitrobenzene (14):** 1,4-Dibromo-2-fluorobenzene (25.00 g, 98.46 mmol) was dissolved in DCM (75 mL), trifluoroacetic acid (75 mL) and trifluoroacetic anhydride (37.5 mL) in a flask and then cooled to 0 °C. To this mixture, ammonium nitrate (9.70 g, 121.17 mmol) was added slowly and the mixture was stirred overnight at RT. The reaction contents were put into ice, deionized water was added and the product extracted with DCM. The organic layer was separated, dried over anhydrous MgSO_4_, filtered and the solvent evaporated to obtain the product. It was purified by recrystallization from EtOH to yield 14 as yellow crystals (25.00 g, 84 mmol, 85% yield) [43]. ^1^H NMR (CDCl_3_, δ): 8.20 (d, 1H, *J* = 6.5 Hz), 7.55 (d, 1H, *J* = 7.5 Hz). ^13^C NMR (CDCl_3_, δ): 161.9, 159.3, 130.9 (d, *J* = 2.0 Hz), 122.9 (d, *J* = 27.0 Hz), 114.9 (d, *J* = 9.0 Hz), 108.9 (d, *J* = 23.0 Hz). ^19^F NMR (CDCl_3_, δ): −97.2 (t, *J* = 7.0 Hz). FT-IR (cm^−1^): 3092, 3017, 2865, 1762, 1584, 1464, 1286, 1222, 1070. EI-MS (*m*/*z*): 298.8 [M]^+^. EA (%) calculated for C_6_H_2_O_2_FNBr_2_: C, 24.11; N, 4.69; Br, 53.47; H, 0.67. Found: C, 24.13; N, 4.72; Br, 52.99; H, 1.06.

**Synthesis of 4,4′-dibromo-5,5′-difluoro-2,2′-dinitrobiphenyl (15):** 14 (19.00 g, 63.56 mmol) and Cu powder (5.32 g, 83.71 mmol) were combined in a flask. The mixture was evacuated and refilled with argon for three cycles before anhydrous DMF (230 mL) was added and heated at 125 °C for 3 h. The reaction contents were cooled to RT and dissolved in toluene and filtered. NaHCO_3_ solution was added to the filtrate and the mixture extracted with toluene and the organic phases combined and washed with deionized H_2_O several times until it became neutral. The organic phase was dried over anhydrous MgSO_4_, filtered and the solvent evaporated to afford the product which recrystallized from isopropanol to yield 15 as yellow crystals (10.63 g, 48.5 mmol, 76% yield) [56]. ^1^H NMR (CDCl_3_, δ): 8.57 (d, 2H, *J =* 6.0 Hz), 7.09 (d, 2H, *J* = 8.0 Hz). ^13^C NMR (CDCl_3_, δ): 161.7 (d, *J* = 258.0 Hz), 142.9, 134.1 (d, *J* = 8.5 Hz), 131.2 (d, *J* = 2.0 Hz), 118.3 (d, *J* = 25.5 Hz), 110.4 (d, *J* = 23.0 Hz). ^19^F NMR (CDCl_3_, δ): –95.9 (t, *J* = 6.5 Hz). FT-IR (cm^−1^): 3109, 3056, 2861, 1790, 1613, 1563, 1488, 1464, 1396, 1293, 1233, 1191, 1066. EI-MS (*m*/*z*): 400.1 [M-2F]^+^. EA (%) calculated for C_12_H_4_O_4_F_2_N_2_Br_2_: C, 32.91; N, 6.40; Br, 36.49; H, 0.92. Found: C, 32.91; N, 6.44; Br, 36.16; H, 1.25.

**Synthesis of 4,4′-dibromo-5,5′-difluorobiphenyl-2,2′-diamine (16):** 15 (10.50 g, 23.97 mmol), ethanol (145 mL) and HCl (45 mL, 35%) were added to a flask. To this mixture, Sn powder (22.76 g, 191.72 mmol) was added over 10 min and refluxed for 90 min. The flask cooled to RT and the mixture filtered and deionized water was added to the filtrate. NaOH solution was added dropwise until the pH became approximately 9. The mixture was extracted with Et_2_O and the organic phase was washed with NaCl, dried over MgSO_4_ and then filtered. The solvent concentrated to obtain 16 as a brown solid (7.34 g, 19 mmol, 81% yield) [57]. ^1^H NMR (CDCl_3_, δ): 6.99 (d, 2H, *J =* 6.0 Hz), 6.90 (d, 2H, *J* = 8.5 Hz), 3.74 (bs, 4H). ^13^C NMR (CDCl_3_, δ): 152.5 (d, *J* = 237.5 Hz), 141.0 (d, *J* = 2.0 Hz), 112.8 (d, *J* = 5.5 Hz), 119.7, 118.0 (d, *J* = 23.0 Hz), 109.4 (d, *J* = 22.0 Hz). ^19^F NMR (CDCl_3_, δ): −121.2 (t, *J* = 7.0 Hz). FT-IR (cm^−1^): 3436, 3411, 3304, 3194, 1620, 1481, 1293, 1194, 1056. EI-MS (*m*/*z*): 377.9 [M]^+^. EA (%) calculated for C_12_H_8_Br_2_F_2_N_2_: C, 38.13; N, 7.41; Br, 42.28; H, 2.13. Found: C, 37.90; N, 7.09; Br, 45.95; H, 2.37.

**Synthesis of 3,6-difluoro-2,7-dibromo-*9H*-carbazole (17):** 16 (6.60 g, 17.45 mmol) and H_3_PO_4_ (150 mL, 85%) were combined in a flask and the mixture was heated at 190 °C for 24h. The flask cooled to RT, and was filtered and washed with deionized H_2_O. Subsequently, the precipitate was dissolved in toluene, passed through flash column chromatography and washed with toluene. The solvent concentrated to afford the product which purified via recrystallization from (10:1, toluene: hexane) to afford 17 as ivory crystals (5.00 g, 14 mmol, 79% yield) [58]. ^1^H NMR (CD_3_COCD_3_, δ): 10.67 (bs, 1H); 8.09 (d, 2H, *J* = 9.0 Hz); 7.85 (d, 2H, *J* = 6.0 Hz). ^13^C NMR (CD_3_COCD_3_, δ): 152.9 (d, *J* = 235.0 Hz), 137.8, 122.2 (dd, *J* = 4.0 Hz, 8.5 Hz), 115.5, 107.2 (d, *J* = 5.5 Hz), 107.0 (d, *J* = 4.0 Hz). ^19^F NMR (CD_3_COCD_3_, δ): −120.3 (dd, *J* = 5.5 Hz, 8.5 Hz). FT-IR (cm^−1^): 3450, 3046, 1694, 1613, 1574, 1474, 1347, 1283, 1205, 1176, 1038. EI-MS (*m*/*z*): 360.9 [M]^+^. EA (%) calculated for C_12_H_5_Br_2_F_2_N: C, 39.93; N, 3.88; Br, 44.27; H, 1.40. Found: C, 40.81; N, 3.87; Br, 43.59; H, 1.83.

**Synthesis of heptadecan-9-ol (18):** In a flask, Mg (6.68 g, 274.84 mmol) was added and heated under high vacuum at 150 °C for 1 h. The flask cooled to RT and was degassed under argon before anhydrous THF (130 mL) and few crystals of iodine were added and the mixture stirred until the colour of the mixture changed from brown to colourless. The flask was cooled to 0 °C before 1-bromooctane (47.92 g, 248.18 mmol) in anhydrous THF (75 mL) was added dropwise. After the addition was completed, the colour of the solution became grey and the mixture refluxed for 2 h at 60 °C to form *n*-octylmagnesium bromide. To this Grignard reagent, ethyl formate (6.13 g, 82.81 mmol) in anhydrous THF (140 mL) was added dropwise at –78 °C during 2 h. The reaction contents were stirred at RT overnight. Methanol and saturated NH_4_Cl solution was added, the product was extracted with diethyl ether and the organic layer was separated and washed with brine solution. The organic layer was separated, dried over anhydrous MgSO_4_ and filtered and the solvent evaporated to afford the 18 as a white solid (21.20 g, 82 mmol, 99% yield) [59]. ^1^H NMR (CDCl_3_, δ): 3.64–3.56 (m, 1H), 1.54–1.25 (m, 28H), 0.90 (t, 6H, *J* = 7.0 Hz). ^13^C NMR (CDCl_3_, δ): 72.0, 37.5, 31.9, 29.7, 29.6, 29.3, 25.7, 22.7, 14.1. FT-IR (cm^−1^): 3507-3021 (broad), 2953, 2918, 2854, 1467, 1379, 1130, 1088. EI-MS (*m*/*z*): 256.3 [M]^+^. EA (%) calculated for C_17_H_36_O: C, 79.61; H, 14.15. Found: C, 79.94; H, 13.63.

**Synthesis of 9-heptadecane-*p*-toluenesulfonate (19):** 18 (20.00 g, 77.98 mmol), triethyl amine (27.1 mL, 194.29 mmol), trimethylammonium monohydrochloride (7.43 g, 77.74 mmol) and DCM (100 mL) were combined in a flask and cooled to 0 °C. To this mixture, *p*-toluenesulfonyl chloride (22.26 g, 116.75 mmol) in DCM (100 mL) was added dropwise during 1h and then the mixture was stirred for 3h. Deionized water was added and the product was extracted with DCM. The organic layer was separated and washed with deionized H_2_O and then with brine. The organic layer dried over anhydrous MgSO_4_, filtered and the solvent evaporated to afford the product. It was purified via column chromatography using (90:10, petroleum ether: ethyl acetate) to obtain 19 as a colourless oil which changed to white crystals within time (28.43 g, 69 mmol, 89% yield) [60]. ^1^H NMR (CDCl_3_, δ): 7.81 (dd, 2H, *J* = 1.5 Hz, 6.5 Hz); 7.34 (dd, 2H, *J* = 0.5 Hz, 8.5 Hz); 4.62–4.51 (qt, 1H, *J* = 6.0 Hz); 2.47 (s, 3H); 1.69–1.51 (m, 4H); 1.38–1.11 (m, 24H); 0.89 (t, 6H, *J* = 7.0 Hz). ^13^C NMR (CDCl_3_, δ): 144.3, 134.8, 129.6, 127.7, 84.6, 34.1, 31.8, 29.7, 29.3 (d, *J* = 7.5 Hz), 29.2, 24.7, 22.7, 21.6, 14.1. FT-IR (cm^−1^): 2953, 2925, 2854, 1602, 1467, 1354, 1169. EI-MS (*m*/*z*): 433 [MNa]^+^. EA (%) calculated for C_24_H_42_O_3_S: C, 70.20; S, 7.81; H, 10.31. Found: C, 70.35; S, 7.44; H, 10.03.

**Synthesis of 3,6-difluoro-*N*-9′-heptadecanyl-2,7-dibromocarbazole (20):** 17 (2.00 g, 5.54 mmol) and KOH (1.76 g, 31.37 mmol) were combined in a flask and then degassed under argon before anhydrous DMSO (40 mL) was added. To this mixture, 19 (3.72 g, 9.05 mmol) was dissolved in anhydrous THF (40 mL) added dropwise and the resulting mixture was heated at 45 °C overnight. The mixture cooled to RT and deionized water was added. The product was extracted with *n*-hexane and washed with saturated solution of brine. The organic phase was separated, dried over anhydrous MgSO_4_ and filtered. The solvent evaporated to afford the product which purified via column chromatography using petroleum ether to obtain 20 as white crystals (2.51 g, 4 mmol, 76% yield) [61]. ^1^H NMR (CDCl_3_, δ): 7.83–7.69 (bm, 2H); 7.58 (d, 2H, *J* = 4.0 Hz), 4.45–4.34 (m, 1H), 2.57–2.11 (bm, 2H), 1.97–1.84 (m, 2H), 1.39–1.07 (m, 20H), 1.04–0.91 (m, 4H), 0.85 (t, 6H, *J* = 7.0 Hz). ^13^C NMR (CDCl_3_, δ): 153.1, 139.5, 135.8, 122.7, 121.2, 115.6, 113.4, 107.6, 106.7, 57.3, 33.5, 31.7, 29.3, 29.1, 26.7, 22.6, 14.1. ^19^F NMR (CDCl_3_, δ): −118.8 (d, *J* = 196.5 Hz). FT-IR (cm^−1^): 2957, 2918, 2850, 1701, 1598, 1467, 1336, 1244, 1191, 1038. EI-MS (*m*/*z*): 599.1 [M]^+^. EA (%) calculated for C_29_H_39_Br_2_F_2_N: C, 58.11; Br, 26.66; N, 2.34; H, 6.56. Found: C, 58.33; Br, 26.01; N, 2.29; H, 6.66.

**Synthesis of 3,6-difluoro-*N*-9′-heptadecanyl-2,7-bis(4,4,5,5-tetramethyl-1,3,2-dioxaborolan-2-yl)carbazole (M4):** 20 (1.00 g, 1.66 mmol), bis(pinacolato)diboron (1.48 g, 5.82 mmol), potassium acetate (0.98 g, 9.98 mmol) and Pd(dppf)Cl_2_ (0.08 g, 0.10 mmol) were combined in a flask and then the system was degassed under argon. To this mixture, anhydrous DMF (20 mL) was added and heated at 100 °C for 48h. The flask cooled to RT and the product was extracted with Et_2_O and the organic phases were washed with deionized H_2_O. The organic layers were separated and dried over anhydrous MgSO_4_, filtered and the solvent evaporated to obtain a product which recrystallized from methanol which passed through the basic alumina to remove the acidic protons to obtain M4 as brown crystals (0.79 g, 1 mmol, 69% yield) [62]. ^1^H NMR (CDCl_3_, δ): 7.89 (bs, 1H); 7.75 (bs, 1H); 7.66 (bd, 2H, *J* = 7.0 Hz), 4.71–4.56 (m, 1H), 2.38–2.23 (m, 2H), 2.02–1.87 (m, 2H), 1.43 (s, 24H), 1.33–1.08 (m, 20H), 1.04–0.90 (m, 4H), 0.84 (t, 6H, *J* = 7.0 Hz). ^13^C NMR (CDCl_3_, δ): 160.8, 139.6, 136.0, 126.4, 124.9, 119.0, 116.5, 114.8, 106.1, 83.9, 56.6, 33.8, 31.7, 29.4, 29.3, 29.2, 26.6, 24.9, 22.6, 14.0. ^19^F NMR (CDCl_3_, δ): −116.28 (d, *J* = 163.0 Hz). FT-IR (cm^−1^): 2978, 2925, 2854, 1609, 1566, 1442, 1332, 1293, 1141, 1066. EI-MS (*m*/*z*): 693.5 [M]^+^. EA (%): calculated for C_41_H_63_B_2_NO_4_F_2_: C, 71.00; N, 2.02; H, 9.16. Found: C, 70.94; N, 2.05; H, 8.90.

**9-(Heptadecan-9-yl)-2,7-bis(4,4,5,5-tetramethyl-1,3,2-dioxaborolan-2-yl)carbazole (M3):** M3 prepared by H. Yi in Iraqi group [33].

**Synthesis of poly[*N*-9′-heptadecanyl-2,7-carbazole-*alt*-5,5-(4′,7′-bis(2-thienyl)-2′,1′,3′-benzothiadiazole-5,6-*N*-(3,7-dimethyloctyl)-dicarboxylic imide)](PCDTBTDI-DMO):** M1 (150 mg, 0.224 mmol) and M3 (147 mg, 0.224 mmol) were added to a flask and degassed under argon. Anhydrous THF (10 mL) followed by sodium hydrogen carbonate solution (2.5 mL, 5% wt, degassed) was added and the system degassed again. To this mixture, Pd(OAc)_2_ (3.7 mg, 0.0168 mmol) and P(*o*-tol)_3_ (10.2 mg, 0.0336 mmol) were added, degassed and heated at 90 °C for 30 h. The flask cooled to RT, the polymer was dissolved in CHCl_3_ (200 mL) and an NH_4_OH solution (50 mL, 35% in H_2_O) added and the mixture was stirred overnight. The organic phase was separated and washed with deionized H_2_O. The organic phase concentrated to around (50 mL) and was put into methanol (300 mL) and stirred overnight. The mixture was filtered and the polymer cleaned using Soxhlet extraction with methanol (300 mL), acetone (300 mL), hexane (300 mL) and then toluene (300 mL). The toluene fraction concentrated to around (50 mL) and was then put into methanol (300 mL). The mixture was stirred overnight and the pure polymer was recovered by filtration to afford PCDTBTDI-DMO as purple powders (150 mg, 0.16 mmol, 73% yield)^33^. GPC: toluene fraction, *M*_n_ = 12,200 g mol^−1^, *M*_w_ = 30,400 g mol^−1^, PDI = 2.4 and Dp = 13. ^1^H NMR (toluene fraction) (C_2_D_2_Cl_4_, δ): 8.12 (d, 2H, *J* = 8.0 Hz), 8.08 (s, 2H), 7.89–7.79 (bm, 2H), 7.63 (d, 2H, *J* = 8.0 Hz), 7.56 (s, 2H), 4.78–4.61 (bm, 1H), 3.90–3.72 (bm, 2H), 2.48–2.30 (bm, 2H), 2.16–2.00 (bm, 2H), 1.85–1.72 (bm, 1H), 1.66–1.48 (bm, 3H), 1.42–1.27 (bm, 6H), 1.24–1.13 (bm, 24H), 1.02 (d, 3H, *J* = 6.0 Hz), 0.88 (d, 6H, *J* = 6.0 Hz), 0.80 (t, 6H, *J* = 6.0 Hz). FT-IR (cm^−1^): 2950, 2925, 2854, 1754, 1701, 1598, 1425, 1336, 1219, 1176, 1066. EA (%) calculated for C_55_H_66_N_4_O_2_S_3_: C, 72.49; H, 7.30; N, 6.15; S, 10.55. Found: C, 70.83; H, 6.98; N, 5.92; S, 10.38.

**Synthesis of poly[*N*-9′-heptadecanyl-2,7-carbazole-*alt*-5,5-(4′,7′-bis(2-thienyl)-2′,1′,3′-benzothiadiazole-5,6-*N*-octyl-dicarboxylic imide)](PCDTBTDI-8):** PCDTBTDI-8 was prepared following the same procedure for synthesis of PCDTBTDI-DMO. M2 (143.2 mg, 0.224 mmol) and M3 (147 mg, 0.224 mmol) were copolymerized for 24h to afford PCDTBTDI-8 as purple powders (170 mg, 0.19 mmol, 86% yield) [33]. GPC: chloroform fraction, *M*_n_ = 20,500 g mol^−1^, *M*_w_ = 65,300 g mol^−1^, PDI = 3.1 and Dp = 23. ^1^H NMR (chloroform fraction) (C_2_D_2_Cl_4_, δ): 8.12 (d, 2H, *J* = 8.0 Hz), 8.08 (s, 2H), 7.89–7.79 (bm, 2H,), 7.63 (d, 2H, *J* = 8.0 Hz), 7.56 (d, 2H, *J* = 3.5 Hz), 4.78–4.61 (bm, 1H), 3.90–3.72 (bm, 2H), 2.48–2.30 (bm, 2H), 2.16–2.00 (bm, 2H), 1.82–1.68 (bm, 2H), 1.45–1.25 (bm, 10H), 1.24–1.11 (bm, 24H), 0.88 (t, 3H, *J* = 7.0 Hz), 0.80 (t, 6H, *J* = 7.0 Hz). FT-IR (cm^−1^): 2921, 2854, 1758, 1701, 1598, 1428, 1336, 1219, 1166, 1095. EA (%) calculated for C_53_H_62_N_4_O_2_S_3_: C, 72.07; H, 7.08; N, 6.34; S, 10.89. Found: C, 70.92; H, 6.96; N, 6.15; S, 10.11.

**Synthesis of poly[*N*-9′-heptadecanyl-3,6-difluoro-2,7-carbazole-*alt*-5,5-(4′,7′-bis(2-thienyl)-2′,1′,3′-benzothiadiazole-5,6-*N*-(3,7-dimethyloctyl)dicarboxylic imide)](P2F-CDTBTDI-DMO):** P2F-CDTBTDI-DMO was prepared following the same procedure for synthesis of PCDTBTDI-DMO. M1 (150 mg, 0.224 mmol) and M4 (155.8 mg, 0.224 mmol) were copolymerized for 48 h to yield P2F-CDTBTDI-DMO as purple powders (214 mg, 0.22 mmol, 98% yield) [33]. GPC: toluene fraction, *M*_n_ = 8700 g mol^−1^, *M*_w_ = 16,300 g mol^−1^, PDI = 1.8 and Dp = 9. ^1^H NMR (toluene fraction) (C_2_D_2_Cl_4_, δ): 8.06 (d, 2H, *J* = 3.5 Hz), 7.86–7.74 (bm, 4H), 7.67 (d, 2H, *J* = 3.5 Hz), 4.63–4.50 (bm, 1H), 3.86–3.70 (bm, 2H), 2.38–2.25 (bm, 2H), 2.09–1.93 (bm, 2H), 1.83–1.69 (bm, 1H), 1.62–1.48 (bm, 3H), 1.45–1.23 (bm, 6H), 1.22–1.12 (bm, 24H), 0.99 (d, 3H, *J* = 6.0 Hz), 0.85 (d, 6H, *J* = 6.0 Hz), 0.78 (t, 6H, *J* = 6.0 Hz). FT-IR (cm^−1^): 2953, 2921, 2854, 1754, 1701, 1556, 1453, 1336, 1176, 1066. EA (%) calculated for C_55_H_64_F_2_N_4_O_2_S_3_: C, 70.19; H, 7.03; N, 5.74; S, 9.86. Found: C, 69.20; H, 6.72; N, 5.79; S, 9.97.

**Synthesis of poly[*N*-9′-heptadecanyl-3,6-difluoro-2,7-carbazole-*alt*-5,5-(4′,7′-bis(2-thienyl)-2′,1′,3′-benzothiadiazole-5,6-*N*-octyl-dicarboxylic imide)](P2F-CDTBTDI-8):** P2F-CDTBTDI-8 was prepared following the same procedure for synthesis of PCDTBTDI-DMO. M2 (143.2 mg, 0.224 mmol) and M4 (155.8 mg, 0.224 mmol) were copolymerized for 24 h to yield P2F-CDTBTDI-8 as purple powders. Toluene fraction (90 mg, 0.10 mmol, 44% yield), chloroform fraction (50 mg, 0.05 mmol, 24% yield) with total yield 68% [33]. GPC: toluene fraction, *M*_n_ = 8200 g mol^−1^, *M*_w_ = 18,400 g mol^−1^, PDI = 2.2 and Dp = 9; chloroform fraction, *M*_n_ = 24,200 g mol^−1^, *M*_w_ = 46,300 g mol^−1^, PDI = 1.9 and Dp = 26. ^1^H NMR (toluene fraction) (C_2_D_2_Cl_4_, δ): 8.06 (d, 2H, *J* = 3.5 Hz), 7.86–7.74 (bm, 4H), 7.67 (d, 2H, *J* = 3.5 Hz), 4.78–4.61 (bm, 1H), 3.90–3.72 (bm, 2H), 2.48–2.30 (bm, 2H), 2.16–2.00 (bm, 2H), 1.82–1.68 (bm, 2H), 1.45–1.25 (bm, 10H), 1.24–1.11 (bm, 24H), 0.88 (t, 3H, *J* = 7.0 Hz), 0.83–0.75 (m, 6H). FT-IR (cm^−1^): 2921, 2854, 1758, 1698, 1566, 1449, 1166, 1045. EA (%) calculated for C_53_H_60_F_2_N_4_O_2_S_3_: C, 69.25; H, 6.58; N, 6.09; S, 10.46. Found: C, 68.15; H, 6.45; N, 5.85; S, 9.98.

## 3. Results and Discussion

### 3.1. Synthesis of Monomers and Polymers

**Scheme 1:**
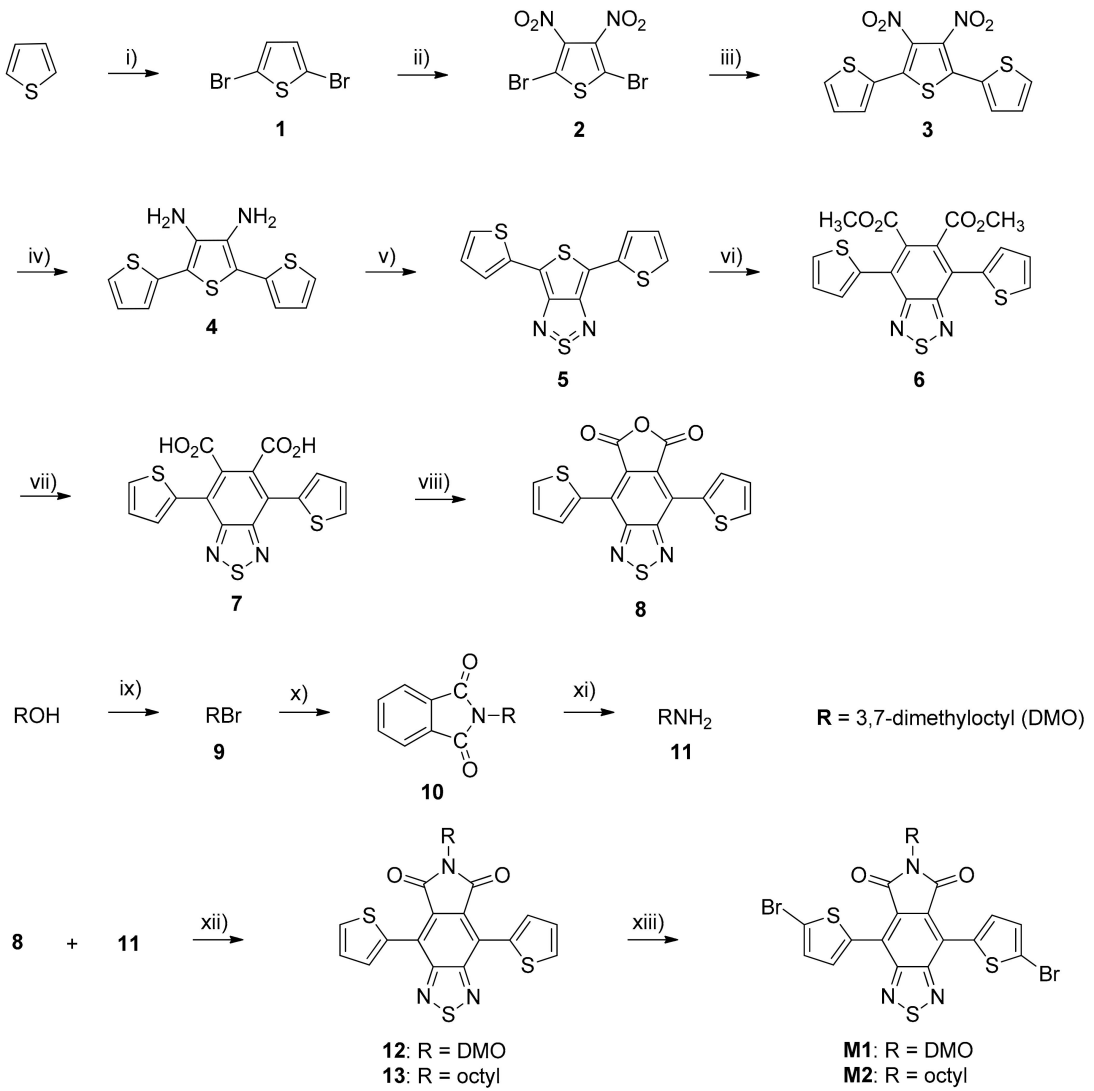
Synthetic steps of the M1 and M2.

The synthetic methods for the preparation of benzothiadiazole dicarboxylic imide (BTDI) monomers (M1 and M2) are outlined in Scheme 1. M1 and M2 were synthesised through several steps starting from commercially available thiophene. For the synthesis of 2,5-dibromothiophene (1), thiophene was selectively brominated at 2,5-positions using two equivalents of *N*-bromosuccinimide (NBS) in DMF in the dark to give 1 as a yellow oily product in a high yield [45]. Then 1 was nitrated with concentrated nitric acid/sulfuric acid and fuming sulfuric acid to give 2,5-dibromo-3,4-dinitrothiophene (2) [46,63,64]. The compound 2 was then reacted with 2-(tributylstannyl)thiophene in the presence of PdCl_2_(PPh_3_)_2_ as a catalyst in anhydrous toluene at 115 °C to give 3′,4′-dinitro-2,2′:5′,2″-terthiophene (3). The resulting compound was obtained as orange crystals in an excellent yield of 90% [47]. 3′,4′-diamino-2,2′:5,2″-terthiophene (4) was obtained by a reduction reaction of 3 using excess anhydrous tin(II) chloride (SnCl_2_). 4 was achieved as a brown solid in an excellent yield of 97% [48]. The resulting substance (4) was then reacted with *N*-thionyl aniline (PhNSO) and trimethylsilyl chloride (TMSCl) in anhydrous pyridine to afford 4,6-bis(2-thienyl)-thieno[3,4-*c*][1,2,5]-thiadiazole (5) as blue crystals in an excellent yield of 93% [49]. The compound 4,7-di(thien-2-yl)-2,1,3-benzothiadiazole-5,6-dimethyl ester (6) was obtained by the Diels–Alder reaction between 5 and dimethyl acetylenedicarboxylate in anhydrous xylene at reflux. It was obtained in an excellent yield of 94% as yellow crystals [50]. Next, the material 6 was hydrolysed under basic conditions in ethanol under reflux followed by acidification to yield 4,7-di(thien-2-yl)-2,1,3-benzothiadiazole-5,6-dicarboxylic acid (7) as a yellow solid in a yield of 85% [51]. The compound 4,7-di(thien-2-yl)-2,1,3-benzothiadiazole-5,6-dicarboxylic anhydride (8) was synthesised by intramolecular ring closure of 7, in the presence of acetic anhydride and anhydrous xylene at 130 °C, as a red solid in an excellent yield of 97% [52]. 3,7-dimethyloctyl bromide (9) was synthesised from the reaction of commercially available 3,7-dimethyl-1-octanol with triphenylphosphine (Ph_3_P)/NBS in dichloromethane as a colourless oil in 73% yield [53]. Then, 9 was reacted with potassium phthalimide in anhydrous DMF to give *N*-(3,7-dimethyloctyl) phthalimide (10) as colourless oil in an excellent yield of 91% [54]. 3,7-dimethyl-1-octanamine (11) was obtained by Gabriel synthesis from the reaction of 10 with hydrazine hydrate (NH_2_NH_2_) in methanol as brown oil in 86% yield [55]. The compounds 4,7-di(thien-2-yl)-2,1,3-benzothiadiazole-5,6-*N*-(3,7-dimethyloctyl)dicarboxylic imide (12) and 4,7-di(thien-2-yl)-2,1,3-benzothiadiazole-5,6-*N*-octyl-dicarboxylic imide (13) were synthesised by the reaction of 8 with 11 and 1-octanamine in the presence of acetic acid and acetic anhydride to yield imide functionalized monomers (12 and 13) as orange solids in 84% and 93% yield, respectively [52]. Lastly, the monomers 4,7-di(5-bromo-thien-2-yl)-2,1,3-benzothiadiazole-5,6-*N*-(3,7-dimethyloctyl)dicarboxylic imide (M1) and 4,7-di(5-bromo-thien-2-yl)-2,1,3-benzothiadiazole-5,6-*N*-octyl-dicarboxylic imide (M2) were prepared by the bromination of 12 and 13 at 5,5′-positions using NBS in THF and to yield M1 and M2 as red solids in excellent yields of 98% and 96%, respectively [51].

Reagents and conditions: (i) NBS, DMF, −15 °C, RT, overnight; (ii) fuming H_2_SO_4_ (20% free SO_3_), conc. H_2_SO_4_, conc. HNO_3_, 20 °C, 20–30 °C, 3 h; (iii) 2-(tributylstannyl)thiophene, anhydrous toluene, PdCl_2_(PPh_3_)_2_, 115 °C, 24 h; (iv) anhydrous SnCl_2_, HCl (35%), ethanol, 30 °C, 24 h, NaOH (25%); (v) PhNSO, TMSCl, anhydrous pyridine, 3 h, RT, HCl (1.0 N); (vi) dimethyl acetylenedicarboxylate, anhydrous xylene, reflux 24 h; (vii) aqueous NaOH, ethanol, reflux 24 h, HCl (35%); (viii) anhydrous Ac_2_O, anhydrous xylene, 130 °C, 6 h; (ix) DCM, PPh_3_, NBS, RT, 90 min; (x) potassium phthalimide, anhydrous DMF, 90 °C, 17 h, KOH; (xi) hydrazine hydrate (51%), methanol, reflux, HCl (5.0 M), reflux, 1 h; (xii) HOAc (100%), 100 °C overnight, Ac_2_O, 100 °C, 6 h; (xiii) NBS, THF, RT, overnight.

**Scheme 2:**
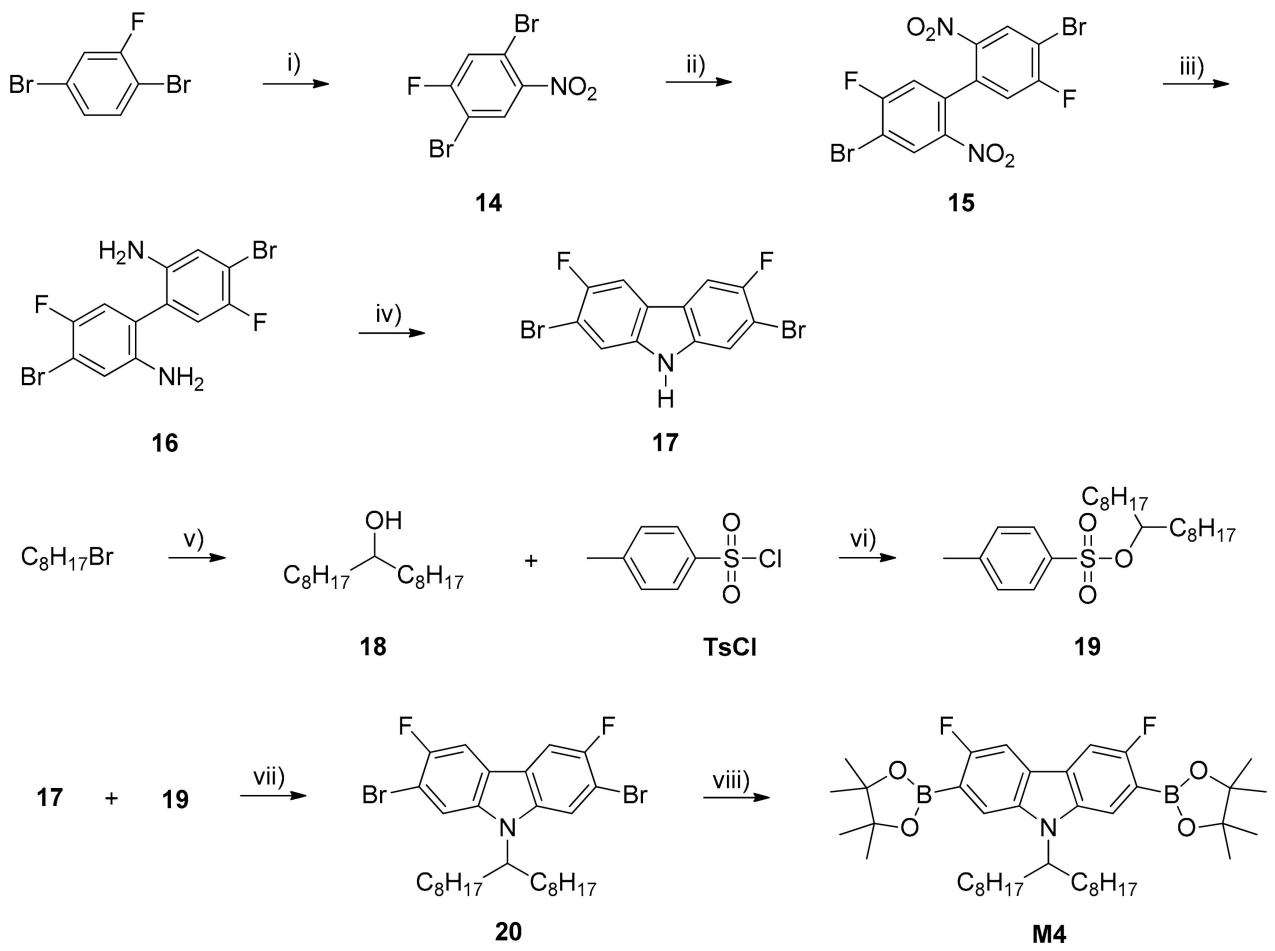
The synthetic steps of the M4.

The monomer 3,6-difluoro-*N*-9′-heptadecanyl-2,7-bis(4,4,5,5-tetramethyl-1,3,2-dioxaborolan-2-yl)carbazole (M4) was synthesised through eight steps starting from commercially available 1,4-dibromo-2-fluorobenzene (Scheme 2). The first step for the synthesis of monomer M4 began with the nitration of 1,4-dibromo-2-fluorobenzene. The reaction was performed between 1,4-dibromo-2-fluorobenzene and ammonium nitrate as a nitrating agent in the presence of trifluoroacetic acid (TFA) and trifluoroacetic anhydride (TFAA) in dichloromethane and to yield 1,4-dibromo-2-fluoro-5-nitrobenzene (14) as yellow crystals in an excellent yield of 85% [43]. 4,4′-dibromo-5,5′-difluoro-2,2′-dinitrobiphenyl (15) was prepared by the Ullmann coupling reaction of the compound 14, using copper powder in anhydrous DMF and 15 was obtained as yellow crystals in a 76% yield [56]. The nitro groups in the compound 15 was reduced using tin powder under acidic conditions in ethanol to obtain 4,4′-dibromo-5,5′-difluorobiphenyl-2,2′-diamine (16) as a brown solid in a yield of 81%^57^. 3,6-difluoro-2,7-dibromo-*9H*-carbazole (17) was prepared by ring closure from 16 using concentrated phosphoric acid and yielded 79% [58]. Heptadecan-9-ol (18) was synthesised through two steps starting from commercially available 1-bromooctane. In the first step, 1-bromooctane was reacted with magnesium metal in anhydrous THF to form *n*-octylmagnesium bromide, which is the Grignard reagent. In the next step, the Grignard reagent was treated with ethyl formate to obtain a secondary alcohol (18) as white solid in an excellent yield of 99% [65]. Then, the compound 9-heptadecane-*p*-toluenesulfonate (19) was prepared by tosylation reaction between 18 and *p*-toluenesulfonyl chloride (TsCl) in the presence of trimethylammonium monohydrochloride/triethyl amine in dichloromethane and yielded 19 as white crystals in 89% yield [60]. The compounds 17 and 19 were reacted under basic conditions in anhydrous DMSO/THF as a co-solvent and yielded 3,6-difluoro-*N*-9′-heptadecanyl-2,7-dibromocarbazole (20) as white crystals in 76% yield [61]. Lastly, the Monomer (M4) was obtained by the reaction of 20 with excess of bis(pinacolato)diboron, potassium acetate base and PdCl_2_(dppf) catalyst in anhydrous DMF to afford M4 as brown crystals in 69% yield [62].

Reagents and conditions: (i) TFA, TFAA, NH_4_NO_3_, DCM, 0 °C; (ii) Cu powder, DMF, 120 °C, 3 h; (iii) Sn, HCl, EtOH, reflux, 3 h; (iv) H_3_PO_4_, 190 °C, 24 h; (v) Mg, THF, ethyl formate; (vi) Et_3_N, Me_3_N.HCl, DCM; (vii) KOH, DMSO, THF, 45 °C, overnight; (viii) bis(pinacolato)diboron, KOAc, anhydrous DMF, Pd(dppf)Cl_2_, 100 °C, 48 h.

**Scheme 3:**
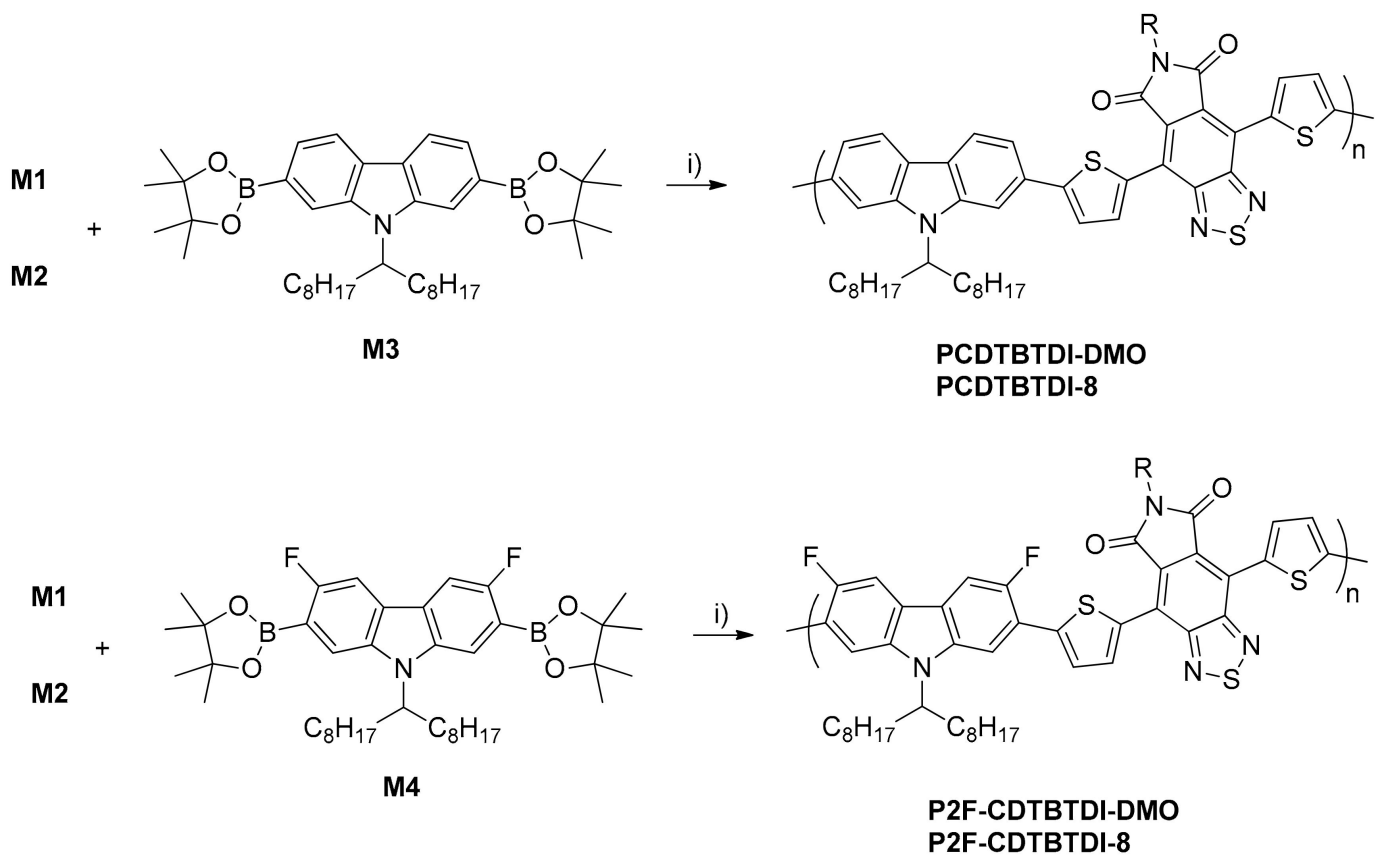
The synthesis of PCDTBTDI-DMO, PCDTBTDI-8, P2F-CDTBTDI-DMO and P2F-CDTBTDI-8 via Suzuki polymerisation. Reagents and conditions: (i) anhydrous THF, NaHCO_3_, Pd(OAc)_2_, P(*o*-tol)_3_, 90 °C, 24–48 h.

The synthesis of the four alternating D-A copolymers, PCDTBTDI-DMO, PCDTBTDI-8, P2F-CDTBTDI-DMO and P2F-CDTBTDI-8 is illustrated in Scheme 3. The polymers were prepared by the Suzuki polymerization between dibromides (M1 and M2) with bis-boronate esters (M3 and M4), respectively. The polymerisations were performed using Pd(OAc)_2_/P(*o*-tol)_3_ catalyst and NaHCO_3_ as a base in anhydrous THF. All polymerisations were left running between 24 and 48 h with large amounts of purple precipitates forming as the reactions proceeded. The polymers were obtained and purified as described in experimental part. The structures of PCDTBTDI-DMO, PCDTBTDI-8, P2F-CDTBTDI-DMO and P2F-CDTBTDI-8 were confirmed by the ^1^H NMR spectroscopy, FT-IR spectroscopy and elemental analysis. The ^1^H NMR spectra of the polymers are available in the supplementary information (See Figures S1–S4).

### 3.2. Molecular Weights and Yield

Molecular weights of the polymers were estimated by GPC using chloroform at 40 °C using polystyrene as calibration standard (Table 1). Substituting 3,7-dimethyloctyl chains in PCDTBTDI-DMO by linear *n*-octyl chains on the BTDI unit yielded PCDTBTDI-8. This polymer gave a chloroform fraction, which has a higher *M*_n_ value relative to PCDTBTDI-DMO. Changing 3,7-dimethyloctyl chains in fluorinated copolymer P2F-CDTBTDI-DMO with *n*-octyl chains in P2F-CDTBTDI-8 on the BTDI moieties results in a polymer with lower *M*_n_ values for the toluene fractions of the polymers. However, P2F-CDTBTDI-8 afforded another fraction in chloroform of a higher *M*_n_ value that was not soluble in toluene, whereas P2F-CDTBTDI-DMO did not provide chloroform fraction. The results indicate that a higher solubility of the polymers with 3,7-dimethyloctyl chains are due to the branching of its substituents. Fluorinated copolymers have lower molecular weights than those of non-fluorinated analogues in toluene fraction. Conversely, P2F-CDTBTDI-8 has the highest *M*_n_ value in chloroform fraction among all polymers prepared. All polymers synthesised in good yield in the range of 68%–98%.

### 3.3. Optical, Electrochemical, Thermal and Structural Study

#### 3.3.1. Optical Properties

**Figure 1:**
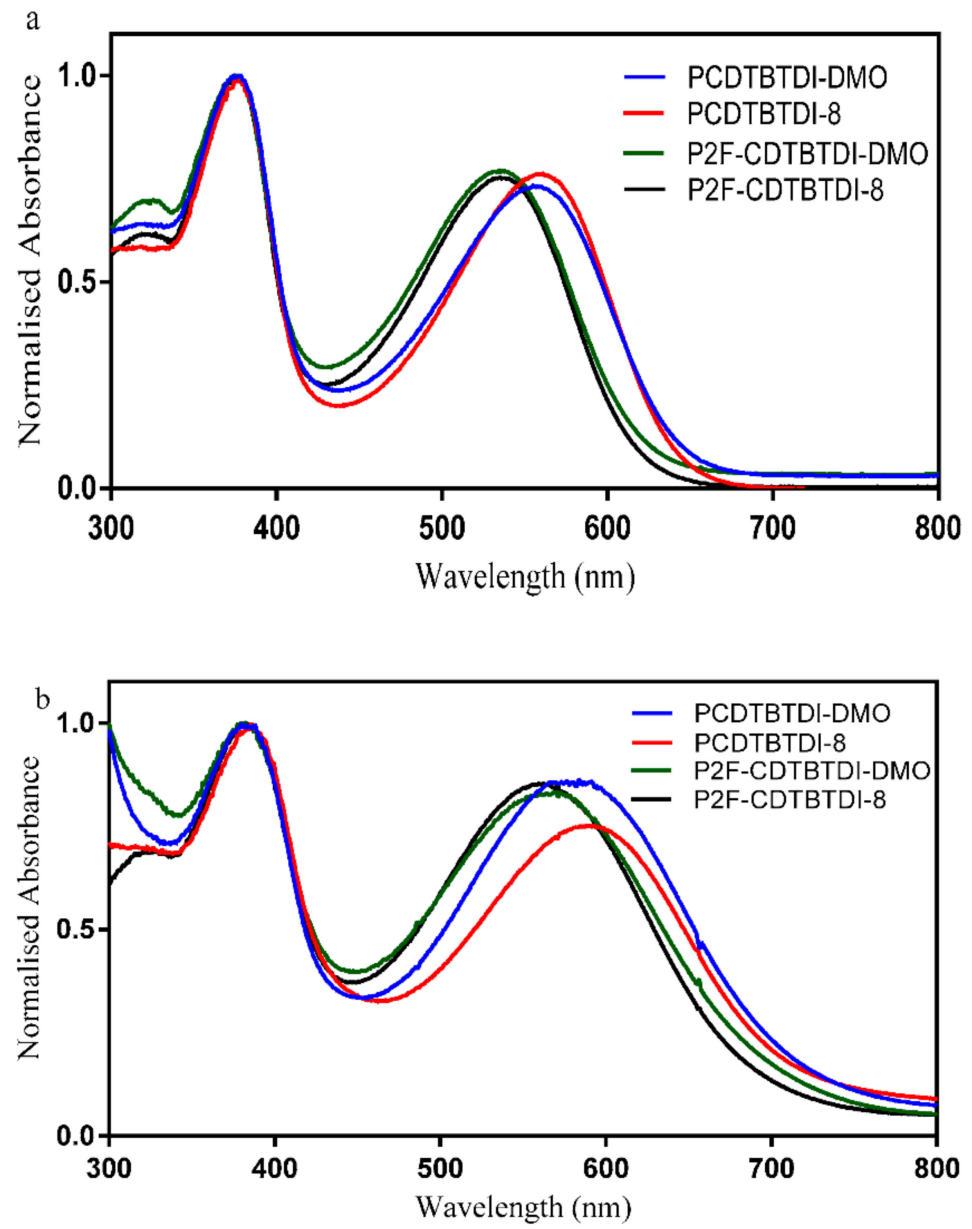
Normalized UV-vis absorption spectra of PCDTBTDI-DMO, PCDTBTDI-8, P2F-CDTBTDI-DMO and P2F-CDTBTDI-8 in (**a**) chloroform solutions; and (**b**) thin-films.

The normalized UV-vis absorption spectra of all polymers were investigated in chloroform solutions and in thin-films (Figure 1 and Table 2). In solutions, the absorption maxima for PCDTBTDI-DMO and PCDTBTDI-8 are 557 nm and 561 nm, respectively. In addition, the same absorption maxima for both P2F-CDTBTDI-DMO and P2F-CDTBTDI-8 is 536 nm. This indicates that the absorption maxima of all polymers is not affected by the nature of substituents (*n*-octyl and 3,7-dimethyloctyl) on BTDI units. In both solutions and films, the fluorinated polymers are blue-shifted relative to those non-fluorinated analogues. This increases the band gap of the fluorinated polymers. This could be explained by the fact that there is a decrease in the intramolecular charge transfer along polymer backbones upon substitution of the carbazole repeat units with electron withdrawing substituents. In thin-films, the absorption spectra of the polymers show a red-shift of absorption bands by 27–32 nm relative to their absorption in solutions. This could be attributed to stronger interchain π-π stacking and more coplanar structures in the solid state. The band gap (E_g_) of the polymers are estimated from the absorption onsets in thin-films. The E_g_ of the PCDTBTDI-DMO and PCDTBTDI-8 are comparable. However, the E_g_ of the P2F-CDTBTDI-DMO and P2F-CDTBTDI-8 are 1.78 and 1.81 eV, respectively. The results indicate that a change of alkyl chains on BTDI units from 3,7-dimethyloctyl chains to *n*-octyl chains does not have any impact on the E_g_ of the PCDTBTDI-DMO and PCDTBTDI-8, while it has a little impact on the E_g_ of the fluorinated polymers.

PCDTBTDI-DMO and PCDTBTDI-8 have lower E_g_ values, around 0.1 eV, relative to those of PCDTBT analogue because BTDI moiety is a stronger acceptor than BT unit [59]. Both polymers (P2F-CDTBTDI-DMO and P2F-CDTBTDI-8) have lower E_g_ compared to the PCffDTBT that was synthesised in the Iraqi group [66]. The absorption coefficient (ε) of PCDTBTDI-DMO is comparable with fluorinated analogue. On the other hand, P2F-CDTBTDI-8 has a lower absorption coefficient than its non-fluorinated counterpart, which may arise from larger E_g_ (Table 2).

#### 3.3.2. Electrochemical Properties

**Figure 2:**
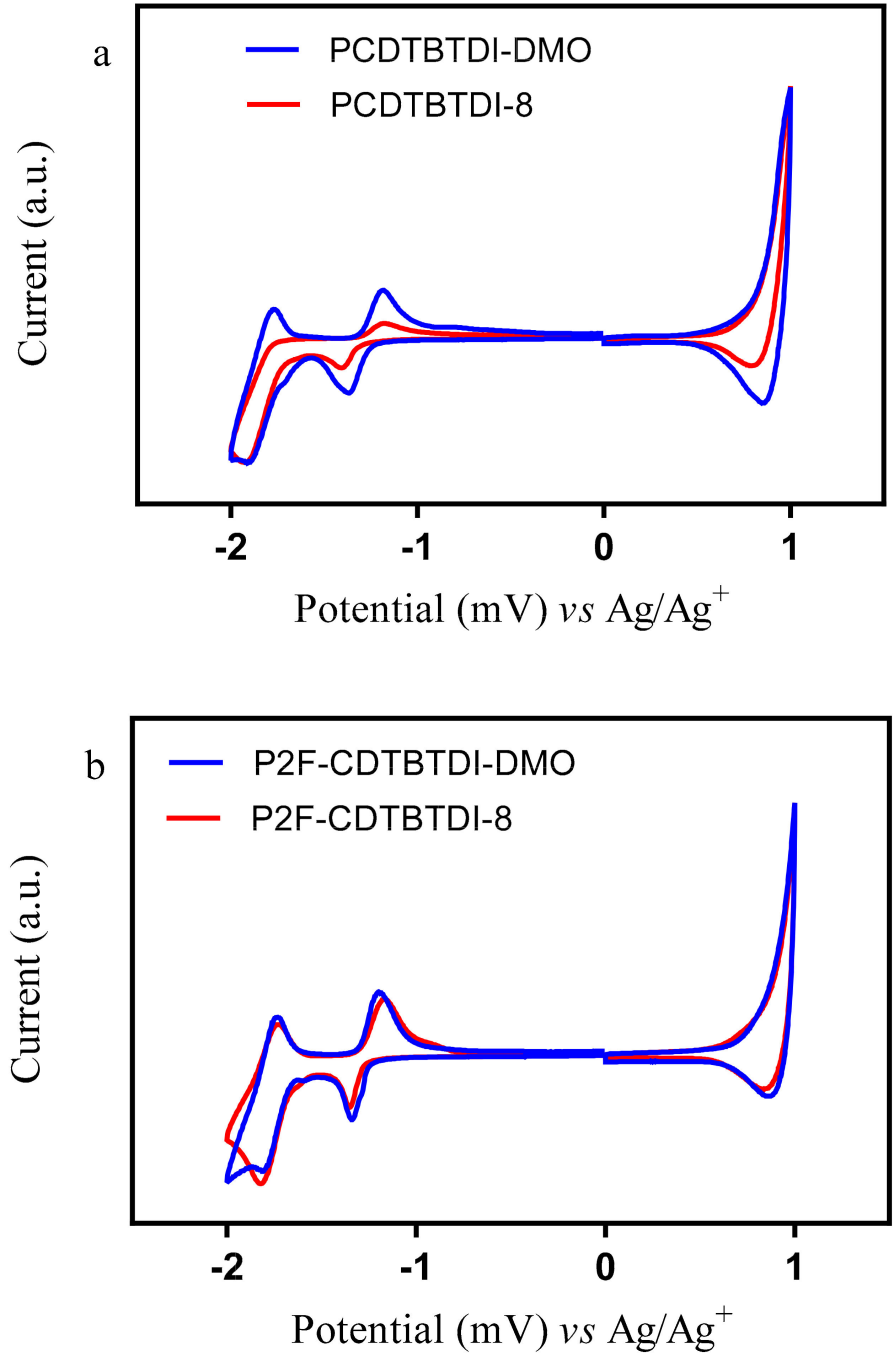
Cyclic voltammograms of (**a**) PCDTBTDI-DMO and PCDTBTDI-8; (**b**) P2F-CDTBTDI-DMO and P2F-CDTBTDI-8 on Pt electrode in Bu_4_NClO_4_/CH_3_CN at 100 mV/s.

The electrochemical properties of the polymers were studied by cyclic voltammetry. All polymers displayed one oxidation wave and two reduction waves at similar potentials. The LUMO and HOMO levels of all polymers were calculated from the onsets of reduction and oxidation potentials, respectively (Figure 2 and Table 3). The onsets were determined from cyclic voltammograms on drop cast polymer films on Pt electrode as the working electrode in Bu_4_NClO_4_/CH_3_CN (0.1 M) vs. Ag/Ag^+^ reference electrode. The HOMO energy levels of all polymers are comparable. The results indicate that the fluorine substituents at the 3,6-positions of 2,7-carbazole units as well as changing the alkyl chains on BTDI units have no impact on the HOMO levels of the resulting polymers. This phenomenon was also observed on other polymers described by Bo and co-workers [43]. However, it is well known that the fluorine substitution can lower the HOMO level of the polymer as reported in previous literature reports [41,61]. All polymers display deep-lying HOMO levels, which are advantageous for the chemical stability of polymers in oxygen. As a result, higher *V*_oc_ values could be expected for the fabricated organic photovoltaic (OPV) devices based on these polymers as donor materials. The LUMO energy levels of all polymers are quite similar because the LUMO energy levels of these materials are dominated by the same BTDI acceptor building blocks. In addition, further anchoring different alkyl chains on BTDI units has little effect on the LUMO energy levels of the polymers.

All polymers have deeper HOMO levels relative to those of O-PDFCDTBT, HD-PDFCDTBT and PCffDTBT analogues [43,44,66]. The HOMO levels of all polymers are comparable to the HOMO level of PCDTBT prepared in Leclerc group (*ca.*−5.5 eV) [59], whereas deeper than the one synthesised in the Iraqi group (−5.35 eV) [33]. The LUMO levels of all polymers are comparable to the LUMO energy level of PCDTBT prepared in Iraqi group (−3.42 eV) [33], and they are higher than LUMO energy level of PCDTBT synthesised in the Leclerc group (*ca.*−3.6 eV) [59].

#### 3.3.3. Thermal Properties

**Figure 3:**
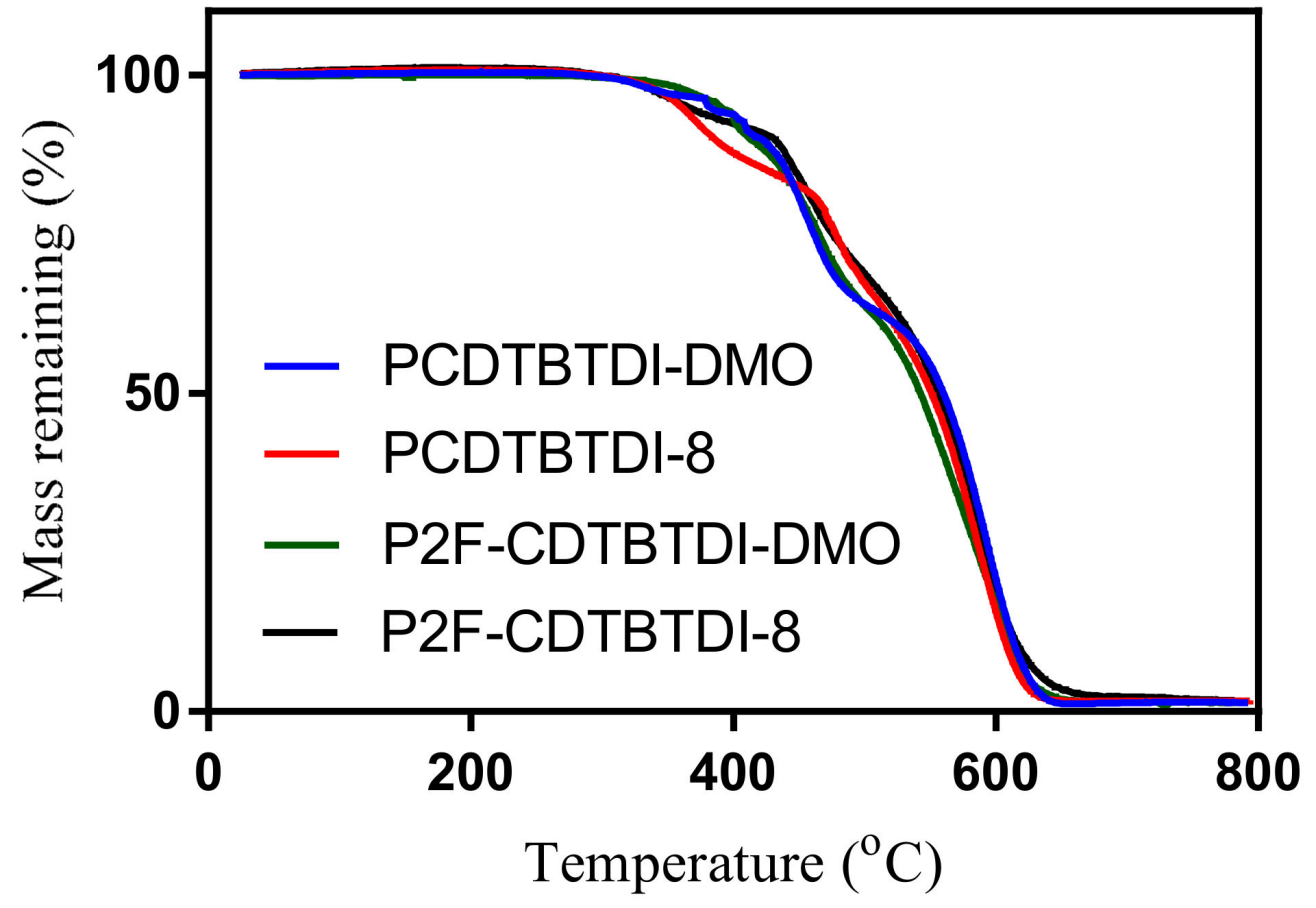
TGA of PCDTBTDI-DMO, PCDTBTDI-8, P2F-CDTBTDI-DMO and P2F-CDTBTDI-8.

The thermal properties of the polymers were studied by TGA (Figure 3 and Table 3). All polymers show high thermal stability with *T*_d_ up to 350 °C. The thermal stability of both fluorinated and non-fluorinated polymers with branched 3,7-dimethyloctyl side chain on BTDI moiety is higher than those with linear *n*-octyl side chain analogues. It can be seen that fluorinated polymers possess higher thermal stability than those non-fluorinated counterparts. This is consistent with previous literatures that exhibit the fluorination at the 3,6-positions of carbazole units, which increases the thermal stability of the resulting polymers [43,44].

#### 3.3.4. Powder X-ray Diffraction

**Figure 4:**
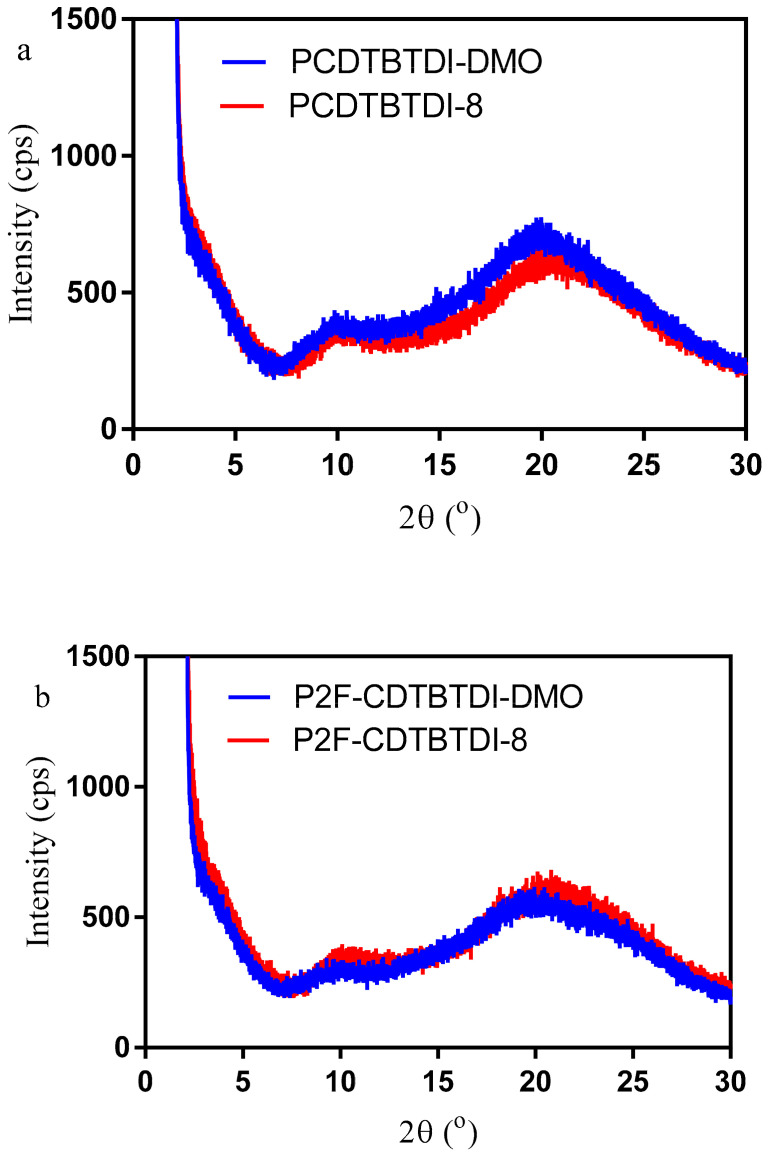
Powder XRD of (**a**) PCDTBTDI-DMO and PCDTBTDI-8; (**b**) P2F-CDTBTDI-DMO and P2F-CDTBTDI-8.

The structural properties of the polymers were studied by powder X-ray diffraction (XRD) in the solid state (Figure 4). The XRD of PCDTBTDI-DMO, PCDTBTDI-8, P2F-CDTBTDI-DMO and P2F-CDTBTDI-8 shows diffraction peaks at 20.2, 19.7, 19.9 and 20.8° corresponding to the π-π stacking distance of 4.39, 4.50, 4.45 and 4.26 Å, respectively. The results show that all polymers have an amorphous nature. The powder XRD of P2F-CDTBTDI-DMO and P2F-CDTBTDI-8 are comparable to the PCffDTBT prepared in the Iraqi group [66]. The results indicate that adding fluorine atoms on the 3,6-positions of carbazole units does not have substantial influence on the crystallinity of the resulting polymers. However, O-PDFCDTBT showed a sharp peak at 5.36° corresponding to a distance of 16.5 Å between conjugated polymer backbones separated by solubilizing alkyl chains [43]. In O-PDFCDTBT, the less bulky linear *n*-octyl chains on the carbazole units adopt more coplanar structure than P2F-CDTBTDI-DMO and P2F-CDTBTDI-8, with bulky branched chains (C17).

## 4. Conclusions

In summary, four novel alternating copolymers PCDTBTDI-DMO, PCDTBTDI-8, P2F-CDTBTDI-DMO and P2F-CDTBTDI-8 were synthesised via Suzuki polymerisation. The polymers were prepared by copolymerising 2,7-linked carbazole units and 3,6-difluoro-2,7-linked carbazole moieties flanked by thienyl units as electron donor units and benzothiadiazole dicarboxylic imide (BTDI) as electron acceptor units. All polymers were prepared in good yields and possess excellent solubility in common organic solvents. Two distinct side chains (linear *n*-octyl vs. branched 3,7-dimethyloctyl) were attached to the BTDI units to investigate the effect of these substituents on the solubilities, molecular weights, optical and electrochemical properties, thermal and structural properties of the resulting polymers. The same properties were investigated when the hydrogen atoms at the 3,6-positions of their 2,7-linked carbazole repeat units were replaced by fluorine atoms. Changing the alkyl chains on BTDI units had an important effect on the solubility of the resulting polymers. The use of branched 3,7-dimethyloctyl chains on BTDI units in the carbazole-based polymers afforded PCDTBTDI-DMO in 73% yield. However, the polymer was extracted in the toluene fraction due to its high solubility. The use of linear *n*-octyl chains on BTDI units yielded PCDTBTDI-8, which has a lower solubility. As a result, the polymer was extracted in chloroform fraction and has a higher *M*_n_ value relative to PCDTBTDI-DMO. In the case of fluorinated copolymers, linear octyl chains had a negative impact on the molecular weight for the toluene fractions of the polymers. Although, P2F-CDTBTDI-8 afforded another fraction in chloroform, which has the highest *M*_n_ value among all polymers prepared. The E_g_ of the fluorinated polymers were slightly changed by replacing 3,7-dimethyloctyl chains with *n*-octyl chains on BTDI units, while the band gaps of the non-fluorinated polymers were comparable. The band gaps of the fluorinated polymers were slightly higher than those of the non-fluorinated analogues. Moreover, the HOMO levels of all polymers were similar. Anchoring different alkyl chains on BTDI units, as well as substituting two hydrogen atoms at the 3,6-positions of the carbazole repeat units by two fluorine atoms, had no impact on the HOMO levels of the polymers. All polymers showed low-lying HOMO energy levels, which are beneficial for the chemical stability of polymers toward oxygen. High *V*_oc_ values could be anticipated for the BHJ photovoltaic cells for these polymers as donor materials and fullerene as acceptor materials. The LUMO levels of the polymers are comparable due to the fact that the LUMO levels of these materials are controlled by the same BTDI acceptor moieties. In addition, attaching different alkyl substituents on BTDI units had little influence on the LUMO energy levels of the polymers. All polymers demonstrated good thermal stability with decomposition temperatures exceeding 350 °C. The thermal stability of the polymers with linear *n*-octyl side chains was lower than those counterparts with branched 3,7-dimethyloctyl side chains on BTDI moieties. Furthermore, the fluorinated polymers have higher thermal stability than those non-fluorinated analogues. The powder XRD of the polymers showed diffraction peaks around 20.0° corresponding to the π-π stacking distance of about 4.0 Å. All polymers have amorphous nature.

## Figures and Tables

**Table 1:** The percentage yield, weight and number average molecular weights with polydispersity indexes of PCDTBTDI-DMO, PCDTBTDI-8, P2F-CDTBTDI-DMO and P2F-CDTBTDI-8.

Polymer	% Yield	Toluene Fraction			Chloroform Fraction		
		*M*_n_ (g mol^−1^)	*M*_w_ (g mol^−1^)	PDI	*M*_n_ (g mol^−1^)	*M*_w_ (g mol^−1^)	PDI
**PCDTBTDI-DMO**	73	12,200	30,400	2.4			
**PCDTBTDI-8**	86				20,500	65,300	3.1
**P2F-CDTBTDI-DMO**	98	8700	16,300	1.8			
**P2F-CDTBTDI-8**	68	8200	18,400	2.2	24,200	46,300	1.9

**Table 2:** Uv-vis data and optical band gaps of the polymers.

Polymer	ε (M^−1^ cm^−1^)	Solution	Film		
		λ_max_ (nm)	λ_max_ (nm)	λ_onset_ (nm)	E_g_ (eV)
**PCDTBTDI-DMO**	31,900	557	583	701	1.76
**PCDTBTDI-8**	29,100	561	588	702	1.76
**P2F-CDTBTDI-DMO**	31,400	536	568	693	1.78
**P2F-CDTBTDI-8**	21,800	536	561	684	1.81

**Table 3:** Thermal and electrochemical properties of the polymers.

Polymer	*T*_d_ (°C)	E_ox_^0^ (V)	HOMO (eV)	E_red_^0^ (V)	LUMO (eV)	E_g (elec)_ (eV)
**PCDTBTDI-DMO**	381	0.84	−5.56	1.23	−3.48	2.08
**PCDTBTDI-8**	359	0.83	−5.55	1.30	−3.41	2.14
**P2F-CDTBTDI-DMO**	392	0.84	−5.56	1.23	−3.48	2.08
**P2F-CDTBTDI-8**	371	0.86	−5.56	1.26	−3.45	2.12

## Supplementary Materials

The following are available online at https://www.mdpi.com/2073-4360/12/12/2910/s1, Figure S1. ^1^H NMR spectrum of PCDTBTDI-DMO in C_2_D_2_Cl_4_ at 100 °C, Figure S2. ^1^H NMR spectrum of PCDTBTDI-8 in C_2_D_2_Cl_4_ at 100 °C, Figure S3. ^1^H NMR spectrum of P2F-CDTBTDI-DMO in C_2_D_2_Cl_4_ at 100 °C, Figure S4. ^1^H NMR spectrum of P2F-CDTBTDI-8 in C_2_D_2_Cl_4_ at 100 °C.
