# Supplementary material for: Influence of Fluorine Substitution on the Optical, Thermal, Electrochemical and Structural Properties of Carbazole-Benzothiadiazole Dicarboxylic Imide Alternate Copolymers
Source: Polymers (Basel). 2020 Dec 4;12(12):2910. doi: 10.3390/polym12122910 (PMC7761964; doi:10.3390/polym12122910)
Supplement: Supplementary file 1 [file polymers-12-02910-s001.pdf]

Supplementary Information

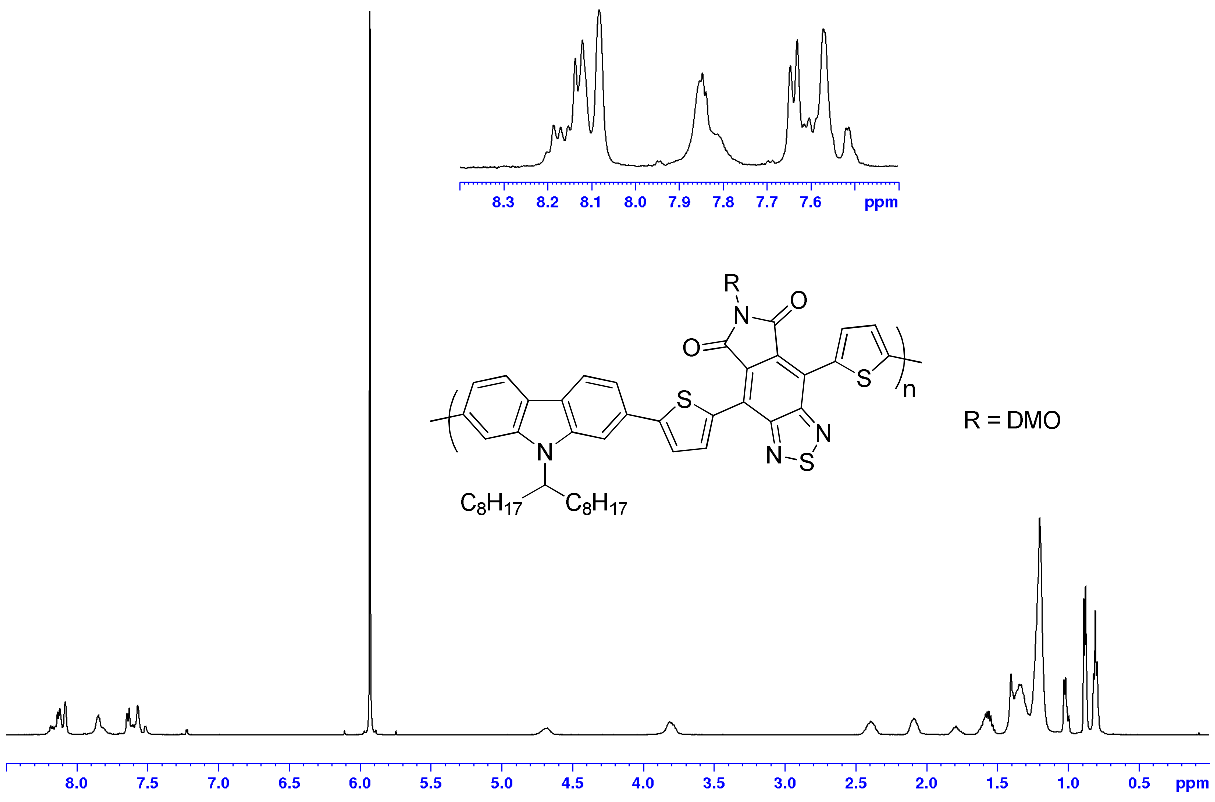

Figure S1. ^{1}H NMR spectrum of **PCDTBTDI-DMO** in $C_2D_2Cl_4$ at 100 °C

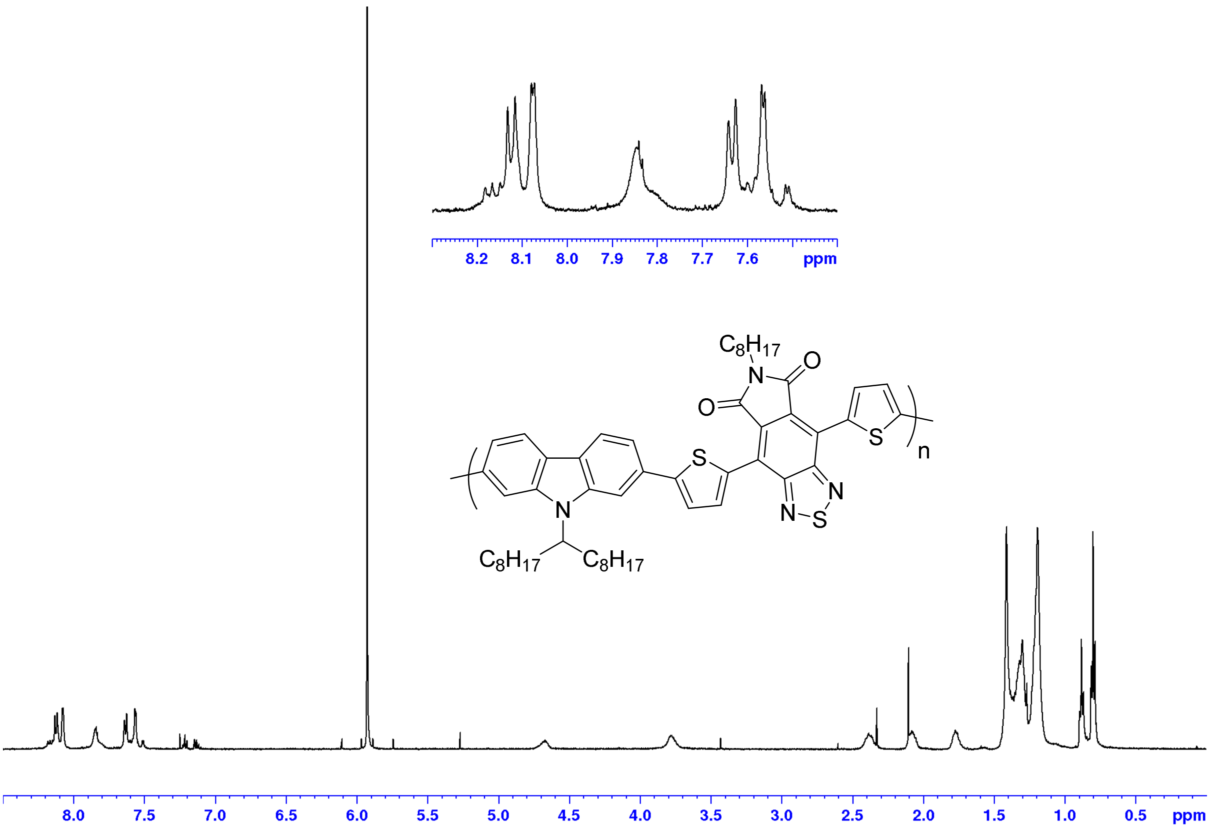

Figure S2. ^{1}H NMR spectrum of **PCDTBTDI-8** in $C_2D_2Cl_4$ at 100 °C

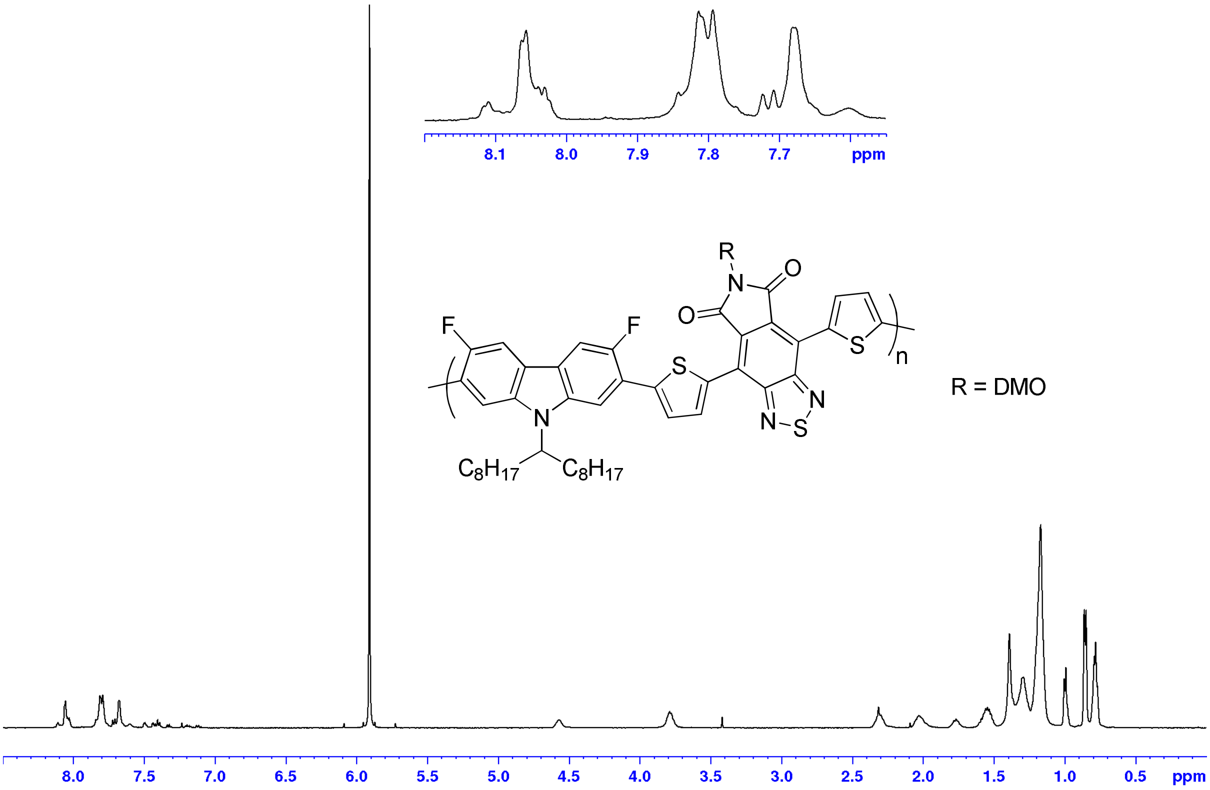

Figure S3. ^{1}H NMR spectrum of **P2F-CDTBTDI-DMO** in $C_2D_2Cl_4$ at 100 °C

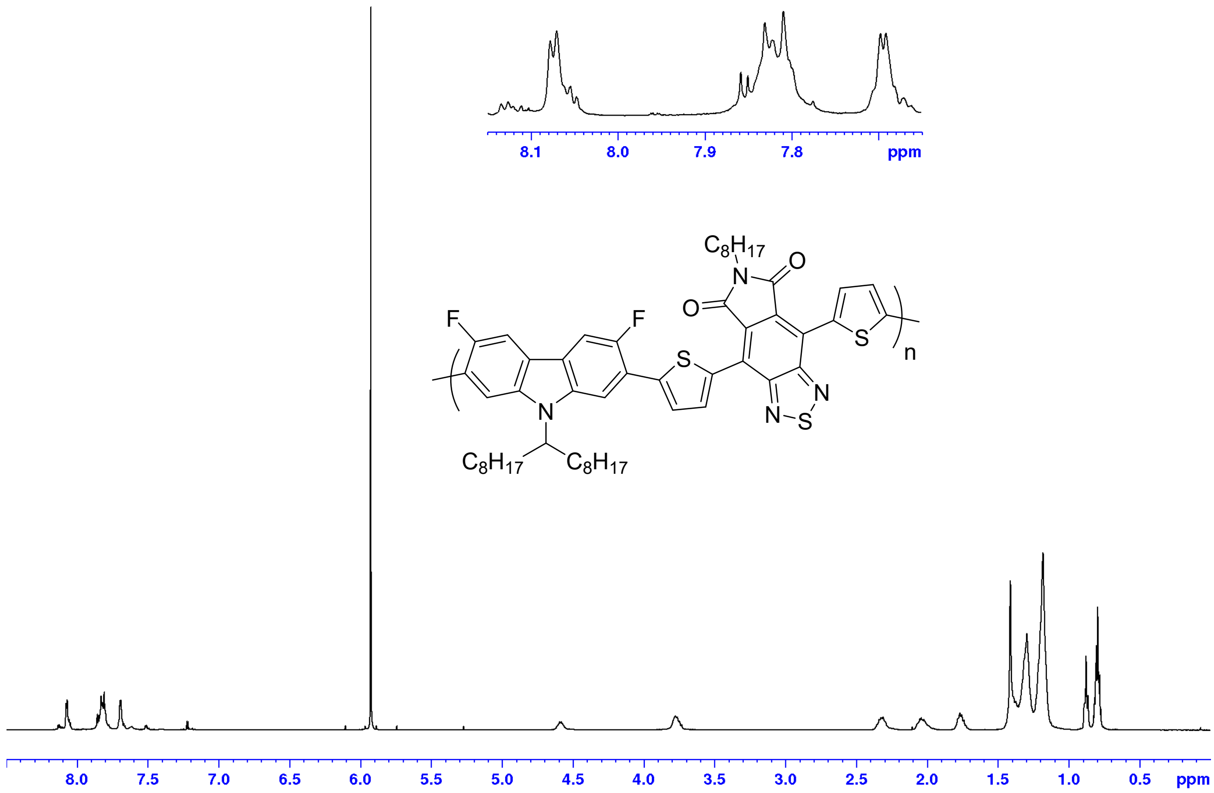

Figure S4. ^{1}H NMR spectrum of **P2F-CDTBTDI-8** in $C_2D_2Cl_4$ at 100 °C
